# Supplementary material for: Universal, school-based interventions to promote mental and emotional well-being: what is being done in the UK and does it work? A systematic review
Source: BMJ Open. 2018 Sep 8;8(9):e022560. doi: 10.1136/bmjopen-2018-022560 (PMC6129100; doi:10.1136/bmjopen-2018-022560)
Supplement: Supplementary file 1 [file bmjopen-2018-022560supp001.pdf]

Search strategy database examples, including MeSH terms
(* indicates truncation of words).

ASSIA

((mental health) OR (mental health intervention OR early intervention)) AND ((school based) OR (scho* OR educat*))) AND (united kingdom OR sco* OR eng* OR northern Ir* OR wales OR wel* OR great britain)) AND (Adolescen* OR child* OR teen* OR youth OR young pe* OR pupil* OR student* OR learner* OR scho*)) AND (Anxi* OR depress* OR resilien* OR emotion* OR stress* OR psycho* OR wellbeing*)) AND (Evaluation OR (pilot OR trial) OR (comparison OR effective)) AND (yr(2000-2016) AND PEER(yes))) AND "SCHOOL BASED" or “SCHOOL”

EMBASE

adolescent health services; adolescent*; anxi*; based; brit*; child*; community mental health services; depress; depress *; early intervention (education); emotion* engl*; health promotion; ir*; kingdom; learner*; mental health; mental health services; northern; pe*; psycho*; pupil*; resilien*; scho*; school; school based; school health services; schools; scot*; stress*; student*; teen*; uk; wales; wel*; wellbeing*; young; young pe*;youth.
