# Supplementary material for: Universal, school-based interventions to promote mental and emotional well-being: what is being done in the UK and does it work? A systematic review
Source: BMJ Open. 2018 Sep 8;8(9):e022560. doi: 10.1136/bmjopen-2018-022560 (PMC6129100; doi:10.1136/bmjopen-2018-022560)
Supplement: Author's manuscript [file bmjopen-2018-022560.draft_revisions.pdf]

BMJ Open is committed to open peer review. As part of this commitment we make the peer review history of every article we publish publicly available.

When an article is published we post the peer reviewers' comments and the authors' responses online. We also post the versions of the paper that were used during peer review. These are the versions that the peer review comments apply to.

The versions of the paper that follow are the versions that were submitted during the peer review process. They are not the versions of record or the final published versions. They should not be cited or distributed as the published version of this manuscript.

BMJ Open is an open access journal and the full, final, typeset and author-corrected version of record of the manuscript is available on our site with no access controls, subscription charges or pay-per-view fees (http://bmjopen.bmj.com).

If you have any questions on BMJ Open's open peer review process please email info.bmjopen@bmj.com

BMJ Open

Universal, school-based interventions to promote mental and emotional wellbeing. What is being done in the UK and does it work? A systematic review

Journal:	*BMJ Open*
Manuscript ID	bmjopen-2018-022560
Article Type:	Research
Date Submitted by the Author:	07-Mar-2018

Complete List of Authors:	Mackenzie, Karen; NHS Ayrshire and Arran, Psychological Services<br>Williams, Christopher; University of Glasgow, Psychiatry

Keywords:	school based, intervention, wellbeing, review, resilience, MENTAL HEALTH

SCHOLARONE™
Manuscripts

For peer review only

Universal, school-based interventions to promote mental and emotional wellbeing. What is being done in the UK and does it work?

A systematic review

Karen Mackenzie*, Chris Williams**

Address for correspondence:
*Psychological Services NHS Ayrshire and Arran
Ayrshire Central Hospital
Kilwinning Road
Irvine KA12 8SS
Email: kazmack2@gmail.com

**Mental Health and Wellbeing
University of Glasgow
1st Floor, Administrative Building,
Gartnavel Royal Hospital
1055 Great Western Road
Glasgow
G12 0XY
E-mail: chris.williams@glasgow.ac.uk

Keywords: Review, school-based, resilience, wellbeing, intervention.

Word count: 5067 (excluding abstract, figures, tables and references).

For peer review only - http://bmjopen.bmj.com/site/about/guidelines.xhtml

ABSTRACT

Objectives: The present review aimed to assess the quality, content and evidence of efficacy of universally-delivered (to all pupils) school-based, mental health interventions designed to promote wellbeing and resilience, provided within the UK.

Setting: A systematic review of published literature set within UK mainstream school settings was conducted.

Participants: 12 studies were included for review (5 primary school based, 7 secondary school based).

Interventions: Selected studies included intervention approaches such as CBT, mental health education, mindfulness and behavioural approaches.

Results: Effectiveness of school-based universal interventions was found to be neutral or small with more positive effects found for poorer quality studies and those based in Primary schools (pupils aged 9-12 years old). Studies varied widely in their use of measures and study design. Methodological issues such as small sample size, varying course fidelity and lack of randomisation reduced overall study quality. Where there were several positive outcomes, effect sizes were small and methodological issues rendered many results to be interpreted with caution. Overall, results suggested a trend whereby higher quality studies reported less positive effects. The only study that conducted a health economic analysis suggested the intervention was not cost-effective.

Conclusions: The current evidence suggests there are neutral to small effects of universal, school-based interventions in the UK that aim to promote emotional or mental wellbeing or the prevention of mental health difficulties. Robust, long-term methodologies need to be pursued ensuring adequate recording of fidelity, the use of validated measures sensitive to mechanisms of change, reporting of those lost to follow up and any adverse effects. Further high quality and large scale research is required across the UK in order to robustly test any long-term benefits for pupils or on the wider educational or health system.

For peer review only - http://bmjopen.bmj.com/site/about/guidelines.xhtml

Limitations:

- Included papers largely based in England so unlikely to be representative of the cultural diversity within UK schools.
- Date limit excluded papers published prior to 2000.

Strengths:

- Addressed a gap in the literature.
- Used a robust methodology to review the literature in this area.
- Conclusions will help inform policy and practice as this topic continues to be debated in current health, education and political spheres.

For peer review only

INTRODUCTION

The mental and emotional wellbeing of children and young people has received increasing attention worldwide. It has been reported that the prevalence of mental health problems ranges from 10-20% [1], and that by the age of 18 up to 20% of young people will have experienced an emotional disorder [2]. Mental health conditions such as anxiety and depression often persist into adulthood [3] and have been associated with a range of negative outcomes including lower academic achievement, higher likelihood of health risk behaviours, self-harm and suicide [4, 5]. However, provision of services for those in need can be as low as 20% [6]. Such access issues to specialist services like CAMHS has meant that school-based interventions have been increasingly explored due to their far reach [7] and existing infrastructure to support child development [8], while noting that schools need support to use the evidence base when applying such interventions [9].

Numerous systematic reviews and meta-analyses have been conducted to review the effectiveness of school-based, mental health interventions at both the universal (delivered to all pupils irrespective of perceived need), and targeted (delivered to vulnerable or 'high risk' individuals only) levels. Overall, this literature has indicated mixed results regarding efficacy of school-based interventions.

For peer review only - http://bmjopen.bmj.com/site/about/guidelines.xhtml

Findings have suggested positive effects on social emotional skills, self-concept, positive social behaviours, conduct problems, emotional distress and problem solving when reviewing school based, universal programs aiming to enhance social and emotional skills [10-11]. Further reviews found Cognitive Behavioural Therapy (CBT) formed the basis of the majority of anxiety prevention programs (78%) and over 75% of trials reported a significant reduction in anxiety [12]. CBT-based interventions were also tentatively endorsed as mildly effective in reducing depression (E.S. = 0.29) and moderately effective (E.S. = 0.50) in reducing anxiety symptoms [13].

With regards to optimal implementation, it has been noted that more positive outcomes were obtained for programs adopting a 'whole-school' approach, that lasted more than one year, and aimed to promote mental health rather than prevent mental illness [11]. A balance of both universal and targeted approaches has been recommended, along with accurate implementation of interventions [14].

However, the long-term impact and target audience of such initiatives has been questioned. A meta-analysis reviewing prevention of depression programmes found that while there was evidence of immediate post-intervention effects, these did not sustain over time (24-36 months) [15]. Moreover, a review evaluating both anxiety and depression programmes found that while the majority were effective for depression (65%) and anxiety (73%), the effect sizes were small (0.12 - 0.29) [16].

It has also been argued that universal prevention interventions are, overall, not efficacious [17-18], with targeted programs being most effective (E.S. = 0.21 to 1.40). Likewise, that while school-based CBT programmes have been demonstrated to lead to a short-term reduction in depression symptoms, interventions are most effective for those in the clinical range [19].

The literature has, therefore, conveyed conflicting results regarding the efficacy of universal school-based interventions whilst consistently in highlighting methodological issues within the existing research base. Common

For peer review only - http://bmjopen.bmj.com/site/about/guidelines.xhtml

issues include a lack of active intervention controls [20], studies' operationalisation and measurements of 'resilience' lack homogeneity [21]; that weak program fidelity and treatment dosage impacts outcomes [10] and that there is insufficient use of validated, standardised measures and long-term follow up [22].

It is also noteworthy that the majority of reviews have focussed worldwide, with most reviewed interventions based in Australia, the US or Canada. No reviews have to date focused on studies in schools in the UK. This trend was also referenced in a NICE funded review [23] of targeted and universal school-based interventions who noted that though findings from international based research are helpful, the generalisability to the UK educational system is questionable. Thus, giving rise to a need for reviews specifically within the UK context.

One systematic review of targeted school-based interventions within the UK research has been conducted [24]. This found that nurture groups demonstrate an immediate positive impact on the social and emotional wellbeing on vulnerable young people, however, that results from longer term follow-up studies are less clear.

The need to carry out a review of universal school-based interventions specifically within the UK context therefore remains. This is especially pertinent in light of the increasing emphasis from national government on developing CAMHS services within the UK, and the impetus on health and education services to work together in order to improve wellbeing outcomes for children and young people [25, 26, 27].

Review aims

The present review aims to fill this gap in the literature by focusing on universally-delivered, school-based mental health interventions provided within the UK only. The following questions will be explored:

For peer review only - http://bmjopen.bmj.com/site/about/guidelines.xhtml

1. How effective are universal school-based interventions in the UK that promote mental health, emotional wellbeing, or psychological resilience and what tools are being used to measure effectiveness?

2. What methodologies are being applied in UK schools when trialling interventions and what is the quality of these studies?

3. What are the intervention characteristics e.g. delivery, content, target audience?

4. What are the identified barriers in delivering and evaluating universal school based interventions?

Search Strategy

Electronic databases were searched for relevant published research on 14th April 2016: EMBASE, CINAHL, MEDLINE, PsycINFO, PsycArticles, ASSIA and Psychological and Behavioural Sciences. Selected journals relevant to the area were hand-searched (British Journal of Educational Psychology; British Journal of School Nursing). Previous reviews and relevant papers were reviewed and following consultation with University librarians, the following keyword search terms were used linked with the Boolean operators 'AND' and 'OR' (* indicates truncation of words):

Mental Health OR Early Intervention OR Anxi* OR depress * OR resilien* OR emotion* OR stress* OR psycho* OR wellbeing

AND

Adolescent OR Adolescen* OR child* OR teen* OR youth OR young pe* OR pupil* OR student* OR learner* OR scho*

AND

School based OR School based mental health OR School based intervention OR interven* OR effect* OR program* OR initiative OR strateg* OR evaluat*

AND

United Kingdom OR UK OR Sco* OR eng* OR northern ir* OR wales OR wel* OR brit* OR kingdom

For peer review only - http://bmjopen.bmj.com/site/about/guidelines.xhtml

Study design criteria was wide to allow for the diverse range of methodologies used to overcome challenges in school-based research. Search terms were, therefore, chosen primarily to promote sensitivity to the subject area. A limit date was set from 2000 to April 2016. The early date limit was selected as this area has been promoted by UK governmental policy largely within the last decade. Further, detailed appraisal of the previous systematic reviews in this area found few, if any, discovered studies prior to this date.

Study selection

The inclusion criteria were as follows:

- The intervention was based in a mainstream school environment;
- Pupils were the recipients of the interventions;
- The study adopted a pre-post design;
- The intervention aimed to target mental health and/or emotional wellbeing;
- The study used a validated measure to quantitatively evaluate emotional or mental wellbeing outcomes and reported those outcomes;
- The study was published between 2000 and April 2016 in a peer reviewed journal.

Exclusion criteria included:

- The study aims or methodology did not fit the inclusion criteria.
- Any studies using a non-validated outcome measure as their primary outcome e.g. Likert scales that were unvalidated.
- Any studies using a purely qualitative methodology.

Details of included and excluded studies (see Figure 1, below.)

Duplicate papers were excluded. Titles were screened to identify only those that clearly met inclusion criteria. Abstracts were assessed independently by the authors. Raters met to compare included papers. Where eligibility was unclear based on the abstract, full articles were retrieved and assessed jointly by raters. Reference lists of included papers were searched as well as previous reviews on related topics. Articles citing included articles were also reviewed and one paper was sourced via this method. Authors of protocol papers were

For peer review only - http://bmjopen.bmj.com/site/about/guidelines.xhtml

contacted leading to an additional paper being sourced. Experts in the field in Scotland, England, Northern Ireland and Wales were contacted regarding any other studies. However, none were eligible for inclusion. Twelve papers were included in the final review.

(Editors: See attached Image)

Quality rating of studies

The Downs and Black [28] checklist was used to assess quality. This checklist assesses internal and external validity, selection bias and study power over 27 items. This checklist was used due to its utility in assessing studies relating to public health and its applicability to assess quality in both randomised and non-randomised studies. Reliability and validity assessment has found the Quality Index to have high internal consistency, good test-retest ($r = 0.88$) and inter-rater ($r = 0.75$) reliability and good face and criterion validity (0.90) [28].

A sample of papers were assessed by an independent researcher (CA). Any rating discrepancies were discussed and a shared decision reached. A decision was taken not to exclude any studies found to be of poor quality as the aim of this current review was to critique universal school-based interventions whilst acknowledging that the real-world implementation of such evaluations can be challenging and, as a result, may reasonably impact study quality.

Data extraction

Due to the heterogeneity of the studies, meta-analysis was not appropriate. A narrative synthesis was applied to explain the findings of this review in line with current guidance [29]. Information gathered from the studies included: study aim, intervention (model, duration, delivery), sample characteristics, study procedures, outcomes and measures, and results. Issues relating to the implementation, as well as effectiveness, of interventions were also noted from those studies commenting on such barriers.

Patient and Public Involvement

No patients or members of the public were directly involved in this piece of research.

For peer review only - http://bmjopen.bmj.com/site/about/guidelines.xhtml

RESULTS

Overview of interventions

Of the twelve studies sourced, five took place in primary schools [30-34] and seven took place in secondary schools [35-41]. An overview of study interventions based in Primary and Secondary schools can be found in Table 1.

Primary school studies:

The five studies within primary school settings evaluated interventions based on computerised CBT [30]; a teacher led intervention embedded within the curriculum (e.g. PATHS – 'Promoting Alternative Thinking Strategies' [31]); manualised anxiety interventions (e.g. a locally developed anxiety intervention, or the Australian developed 'FRIENDS' programme) delivered by both school staff (teachers and nurses) and external health staff (e.g. psychologists) [32-34].

Secondary school studies:

Three of the secondary school-based studies trialled interventions based on Cognitive Behavioural Therapy principles (e.g. UK Resilience programme (UKRP), Resourceful Adolescent Programme (RAP-UK) [36, 39, 41]) delivered by school staff [36], educational psychologists [39] and external facilitators [41]. Interventions were also said to include principles of Interpersonal Therapy (RAP-UK [41]) and behavioural approaches ('TRY' [39]).

One study trialled an intervention based on Positive Psychology [35], two studies trialled a mindfulness-based intervention [38-39] and two trialled locally developed mental health education sessions delivered to all pupils [37,40]. These interventions were led by trained school teachers [35, 38, 40], and trained volunteers [37]. All delivered the intervention during Personal Health and Social Education (PHSE) classes.

Methodological quality

The quality of studies ranged from 'poor' (34% [30]; 37.5% [35]) to 'excellent' (75% [34,37]; 78.1% [36]; 81.3% [41]).

Six studies used a randomised controlled pre-post design [30-32, 34, 37, 41]. The remaining were non-randomised pre-post designs and only one did not

For peer review only - http://bmjopen.bmj.com/site/about/guidelines.xhtml

have a control group [33]. Some studies were particularly weak on their description of sample characteristics and representation of the population [30,35], reporting of those lost to follow up and accounting for those in the analysis [32,35], and the exploring of adverse events, of which only one study provided information [41]. Only six studies provided a power calculation [31, 34,36,37,40,41], most of which had samples sufficiently powered to determine an effect (except [37]). The remaining studies did not provide such information.

Of the eleven studies employing controls, six used controls from the same school in which the intervention was taking place (32,34,36,37,39). All other studies recruited controls from different schools.

Sample sizes ranged from 13 [30] to 5075 [31]. The age of participants ranged from 4 [31] to 16 years old [38,41] with the majority of studies targeting the early adolescent age range (9-12 years old) at the end of Primary school or at the beginning of Junior / Secondary school [30, 32, 34-37].

Table 1. Overview of interventions based in Primary and Secondary schools.

PRIMARY SCHOOLS

Study (Location)	***Sample***	***Study aim / hypothesis***	***Intervention - Theoretical model and Content***	***Intervention - Setting, Structure and Delivery***
Attwood et al., 2012 [30] ***(Bristol, England)***	*10-12 year old boys from two co-educational schools.* *(n=13)*	*A proof of concept study to explore the viability and possible benefits of a cCBT programme.*	*'Think, Feel, Do' - Based on CBT principles with a psychoeducation component. Cartoon characters guide users through various activities including: emotional recognition; linking thoughts, feelings and behaviours; identifying and challenging negative thoughts; and problem solving. Involves quizzes, practical exercises, videos, music and animation.*	*Six x 45min sessions delivered via an interactive multimedia CD-ROM.* *Took place within the school, facilitated by the researcher.*

For peer review only - http://bmjopen.bmj.com/site/about/guidelines.xhtml

Berry et al., 2016 [31] <br>***(Birmingham, England)***	*4-6 year old pupils (n=5075; 56 x schools)*	*Test the effectiveness and cost-effectiveness of the intervention to reduce children's level of behavioural and emotional difficulty.*	*'PATHS' (Promoting alternative thinking strategies). Aims to improve skills in five areas: self-awareness, managing feelings, motivation, empathy and social skills. Lessons are developmentally sequenced and focus on techniques for self-control; emotional and interpersonal understanding steps for solving interpersonal problems; positive self-esteem and improved peer relationships.*	*44 lessons in Year 1; 47 x lessons in Year 2. Delivered by trained teachers within classroom.* <br>*Manual provides teacher scripts, pictures, activity sheets, photos, posters, and home activities.*
Collins et al., 2013 [32] <br>***(South Lanarkshire, Scotland)***	*9-10 year old pupils (n=317; 9 schools; 18 classes).*	*To explore if anxiety & coping showed improvement post-intervention, and test effects of delivery.*	*'Lessons for living: Think Well, Do Well'. CBT based intervention to develop coping skills. A series of skills practice using interactive teaching methods. Children are guided to recognise emotional symptoms, reduce avoidant coping strategies, and focus on proactive problems solving and support-seeking.*	*Ten lessons delivered by a psychologist (n=103) & teacher (n=79) during PSE. Teachers provided with intervention manual following one-day training.*
Stallard et al., 2007 [33] <br>***(Bath & N.E. Somerset, England)***	*9-10 year old pupils (n=106; 3 schools; 4 classes).*	*To evaluate an Australian-originated intervention in the UK; test delivery by school nurses.*	*'FRIENDS - Feelings, Relax, I can do it, Explore solutions, Now reward, Don't forget practice, Smile'. Based on CBT principles it teaches children practice skills to: identify their anxious feelings and learn to relax; identify unhelpful thoughts and replace them with helpful thoughts; face and overcome problems and challenges.*	*Ten sessions delivered by school nurses who attended 2-day training. Lessons comprise group work, workbooks, role play and games. Parents invited to pre-intervention session.*
Stallard et al., 2014 [34] <br>***(Bath, N.E. Somerset, Swindon, Wiltshire, England)***	*9-10 year old pupils (n=1448; 45 x schools)*	*To assess the effectiveness of FRIENDS delivered by both health and school professionals on anxiety prevention.*	*As above (Stallard et al., 2007)*	*Nine x 60 min lessons delivered to whole classes. Health-led group: two trained facilitators. Teacher-led group: led by class teacher. All attended 2-day training.*

For peer review only - http://bmjopen.bmj.com/site/about/guidelines.xhtml

SECONDARY SCHOOLS

Study (Location)	*Sample*	*Study aim / hypothesis*	*Intervention - Theoretical model and Content*	*Intervention - Setting, Structure & Delivery*
Boniwell et al., 2016 [35] ***(S.E. London, England)***	*11-12 year old pupils (n=296; 2 x Haber-dashers' Aske's Fed. of Schools)*	*To test the efficacy of a new school programme for the promotion of happiness and wellbeing skills.*	*'Personal Wellbeing Lesson Curriculum'. Covers the 'scientific basis of happiness' focusing specifically on two core aspects: positive emotions / experiences and positive relationships. Based on theoretical constructs from wellbeing research and positive psychology e.g. 'three good things', forgiveness letter, gratitude visit.*	*Eighteen bi-weekly 50 min scripted lessons delivered to 8 classes by 4 teachers who attended 5-day training. Provided with lesson plans, PowerPoints and handouts.*
Challen et al., 2014 [36] ***(Greater London, N.W. England & N.E. England)***	*11-12 year old pupils (n= 2844; 16 x schools; 3 x L.As)*	*To evaluate a UK version of Penn Resiliency Program (PRP). Hypothesised high completion rates and reduction of depression symptoms.*	*'UK Resiliency Program'. Aims to build resilience and promote realistic thinking and adaptive coping, based on Ellis's 'Activating event-belief consequences model'. Teaches cognitive behavioural and social problem-solving skills; encourages accurate appraisal of situations; and assertiveness, negotiation and relaxation skills.*	*An 18-hour program delivered within the timetable at the teacher's discretion. Delivered by school staff who attended 10-day training in the US.*
Chisholm et al.,2016 [37] ***(Birming-ham, England)***	*12-13 year old pupils (n=769; 6 x schools).*	*To test whether contact with an individual with MH diagnosis plus education is more effective in reducing stigma, improving MH literacy and promoting wellbeing than education alone.*	*'Schoolspace'. A 10-module MH intervention designed by study researchers covering topics such as stress, depression, psychosis, different ways of thinking and a drama workshop. The 'contact' group had an individual facilitating who was a MH service user and had a diagnosis (e.g. psychosis, BPD) - this was revealed halfway through the day.*	*A one-day intervention within the school led by NHS staff, trained volunteers and MH service users.*
Kuyken et al., 2013 [38] ***(England)***	*12-16 year old pupils (n= 522; 12 x*	*To investigate the acceptability of a mindfulness programme for teachers and*	*'Mindfulness in Schools Program' (MiSP). Involved learning to direct attention to immediate experience with open-minded curiosity and acceptance. Skills were learned*	*Nine weekly scripted lessons delivered as part of the curriculum, or at lunchtime by 7*

For peer review only - http://bmjopen.bmj.com/site/about/guidelines.xhtml

	schools).	students; test efficacy of programme on MH and wellbeing.	through practice sessions and everyday application. Mindfulness practice used to work with mental states and everyday stressors to cultivate wellbeing and promote mental health.	teachers trained and approved to deliver the MiSP curriculum.
**Rice et al., 2015 [39]**<br><br>**(S.E. England)**	13-14 year old pupils (n=256; 3 x schools).	To compare three types of intervention which may prevent adolescent depression and explore cognitive mechanisms involved with each.	'TRY' (Thinking about Reward in Young People). Aimed to enhance reward processing through actively selecting activities to lift mood.<br><br>CBT. Aimed to change negative thinking patterns by encouraging evaluation of thoughts.<br><br>'MBCT' (Mindfulness Based Cognitive Therapy). Aimed to promote awareness and acceptance of thoughts and to develop regulation of attention through guided meditation.<br><br>Psychoeducation regarding depression was provided to all groups.	Eight weekly manualised sessions of each intervention delivered within 50-minute PHSE lessons by Educational Psychologists who attended regular supervision.
**Naylor et al. 2009 [40]**<br><br>**(Greater London, England)**	14-15 year old pupils (n=416; 2 x schools).	To explore whether teaching adolescents about mental health would result in gains in knowledge and empathy.	Mental health lessons. Topics included: stress, learning disability, depression, suicide / self-harm, eating disorders, and bullying using methods such as discussion, role playing and internet searching.	Six 50 min weekly lessons delivered by 7 group tutors from pastoral care who attended a 1-day training from researchers.
**Stallard et al., 2013 [41]**<br><br>**(Bath, N.E. Somerset, Bristol, Wiltshire, Notting-hamshire, England)**	12-16 year old pupils (n=5030; 8 x schools, 28 x year groups).	To assess effects of classroom based CBT on symptoms of depression and in relation to other aspects of psychological wellbeing and specific demographic sub-groups.	'RAP-UK: Resourceful Adolescent Programme'. A depression prevention programme based on CBT and interpersonal therapy principles adapted to fit the UK curriculum. Key elements include: personal strengths, helpful thinking, keeping calm, problem solving, support networks and keeping the peace. Students complete workbooks as they progress.	Nine 50-60 min manualised lessons delivered within the PSHE curriculum by two trained facilitators external to the school.<br><br>Two booster sessions offered to schools at 6 month follow up.

For peer review only - http://bmjopen.bmj.com/site/about/guidelines.xhtml

EFFECTIVENESS OF INTERVENTIONS

An overview of study characteristics and outcomes can be found in Tables 2 and 3.

Data collection and measurement

Studies varied widely in their use of measures. Measures used to rate depressive symptoms included the Children's Depression Inventory (CDI) [36], the Short Mood and Feelings Questionnaire (SMFQ) [39, 41] and the Center for Epidemiological Studies – Depression Scale (CES-D) [38]. Measures used to rate anxiety included the Revised Children's Anxiety and Depression Scale (RCADS) [34, 41], Revised Children's Manifest Anxiety Scale (RCMAS) [36], Penn State Worry questionnaire [41] and the Spence anxiety scale [30,32,33]. Measures used to capture different methods of coping related to symptoms of anxiety or depression included: Children's Automatic Thoughts Scale (CATS) [41], Coping Strategy Indicator (CSI) [32], Sentence Completion for Events in the Past Test (SCEPT) [39], and Perceived Stress Scale (PSS) [38]. Two studies used measures related specifically to wellbeing or resilience: Warwick-Edinburgh Mental Wellbeing Scale (WEMWBS) [38] and the Resilience Scale [37] and others used measures related to self-esteem [33,34,41] and life satisfaction [35]. The Strength and Difficulties Questionnaire (SDQ) was the most commonly used measure said to rate behavioural, emotional difficulties and overall functioning and either the child, parent or teacher version was used in six of the twelve studies [30,31,33,36,37,40]. Studies varied according to the length of follow up ranging from 4 weeks [37], to 2 years [41]. Four of the twelve studies sought to obtain qualitative, as well as quantitative data [30,35,37,41]. However, it was beyond the scope of this paper to comment on qualitative findings.

Due to the heterogeneity of studies, the effectiveness of each intervention approach will be discussed in turn. Overall, results suggested a trend whereby higher quality studies reported less positive effects.

Studies trialling bespoke mental health education programmes (n=3; [40,37,35] – all in secondary schools).

Two studies found small (d=0.11-0.22) but significant improvements in total and subscale SDQ scores for those that received mental health education.

For peer review only - http://bmjopen.bmj.com/site/about/guidelines.xhtml

However, of those, it is noteworthy that Chisholm et al. [37] did not employ a non-intervention condition. Boniwell et al. [35] trialled a bespoke intervention based on Positive Psychology principles and found a decrease in outcomes of life satisfaction and an increase in negative affect for both groups. However, this was less so for the intervention group (d=-0.24 compared to d=-0.79) which was interpreted as the intervention having a 'buffering effect' at a time of stress for the pupils.

Studies trialling CBT-based interventions [n= 8; 30-34,36,39,41]. These are described by setting (Primary and then Secondary).

Primary schools

All primary-school based studies trialled interventions pertaining to altering thinking styles based on CBT principles. Four studies, three of which employed a control arm, reported statistically positive outcomes on anxiety-related measures following interventions including FRIENDS [33,34], 'Think Feel Do' [30] and locally developed CBT programmes [32] with larger effects for those in 'high risk' groups (d=-1.26; [33] – no control arm). Methodological issues such as a small sample size and significant group differences at baseline (n=13;[30]), failure to include those lost to follow-up in analysis [32], lack of controls [33], and small effect sizes for universal samples (d=0.01 - 0.2) [34] should be noted when taking inference from those results. Mixed results were found in relation to delivery, with stronger effects found in interventions led by health professionals (d=0.2) versus school staff (d=0.02) [34], or no difference between psychologist or teacher-led interventions [32]. A sufficiently powered, good-quality study evaluating the use of PATHS within the curriculum found few, small significant results (d = 0.06 – 0.14; teacher-rated intervention measure) at 12-month follow up, and no effects on any measure at 24-month follow up [31].

Secondary schools

Fewer significant outcomes were found in trials based within secondary school populations. Small (d=0.093), but short-lived positive outcomes were found on the CDI for those in the UKRP intervention [36]. Mixed results were found for those in the UK-RAP intervention, with results indicating some beneficial and also potentially negative outcomes [41] although all with small

For peer review only - http://bmjopen.bmj.com/site/about/guidelines.xhtml

effect sizes. Both were high quality, longitudinal, well-powered studies employing robust methodologies. Further, no effects were found in the CBT group when compared with as-usual controls or other treatments in a smaller study looking at mechanisms of change [39]. In the same study, a behavioural intervention (TRY) was found to have positive effects on reward-seeking behaviour and SMFQ measure (d=-0.8) when compared with other treatments; however, this finding was not confirmed when compared with PHSE-as-usual controls.

Studies using mindfulness-based interventions (n=2; [38,39] - both in secondary schools).

Positive outcomes were found in a feasibility study evaluating a mindfulness-based intervention [38] which yielded statistically significant, modest effects on both depression (CES-D: d=-0.24) and wellbeing (WEMWBS: d=0.15) measures. Due to small sample sizes this study was likely to be underpowered; however, outcomes were sustained at 3 months follow up and were associated with greater mindfulness practice. No significant outcomes were found in a smaller study trialling MBCT on measures of mood (SMFQ) or reward-seeking [39].

For peer review only - http://bmjopen.bmj.com/site/about/guidelines.xhtml

For peer review only

Table 2. Design and outcome characteristics of primary-school based studies.

Study (% quality rating)	Study Design	Measures	Follow-up	Effects / Outcomes
**Attwood et al., 2012 [30]** (34%)	*Randomised pre-post intervention evaluation using opportunistic sample. No blinding or randomisation procedure reported. 'cCBT' (n=6) x control group (n=7).*	• *SCAS - Parent & Child version.*<br>• *SDQ - Parent version.*<br>• *Focus groups (n=8)*	*Baseline; 6-weeks post intervention.*	*Significant reduction in SCAS-C 'social' (d=0.49*) and 'general anxiety' (d=0.48*) subscales (NB: Intervention group significantly higher on SCAS at baseline). No effects on parent rated measures.*
**Berry et al., 2016 [31]** (68.8%)	*Randomised controlled trial; web randomisation system.*<br><br>*29 schools 'PATHS' intervention x 27 schools WL Control.* [1]	• *SDQ - teacher version.*<br>• *PATHS teacher rating scale (PTRS).*<br>• *T-POT.*	*Baseline; 12 month post-intervention; 24 month post intervention.*	*No differences on SDQ at 12-month F-up. Some significant results on subscales of PTRS at 12 month f-up (Social competence: d=0.09*; Aggression: d=0.14*; Inattention: d=-0.06*; Peer relations: -0.10*). Not maintained at 24 month follow up.*
**Collins et al., 2013 [32]** (46.9%)	*Randomised 3 x 3 mixed design. No randomisation procedure reported.*<br><br>*Psychologist led anxiety intervention (n=103) x Teacher led anxiety intervention (n=79) x Controls (n=135).*	• *CSI*<br>• *SCAS -Child version administered by teachers.*	*Baseline; post-intervention; (within 3 weeks of end); 6 months follow up.*	*Improvement in psychologist-led and teacher-led groups on SCAS-C (d=0.41*; d=0.31*) & CSI 'Avoidance' (d=0.31*; d=0.31*) and 'problem solving' (d=-0.66*; d=0.52*) subscales. No difference between psychologist or teacher-led groups. SCAS-C outcomes maintained at 6 months follow up (d=0.39*; d=0.39*). NB: Those lost to follow up (n=155) not included in analysis.*
**Stallard et al., 2007 [33]** (43.4%)	*Pre- post evaluation of pupils (n=106) from 3 schools taking part in the FRIENDS intervention.*<br><br>*No controls employed.*	• *SCAS-Child version.*<br>• *CFSEQ.*	*'T1': 6 month prior; 'T2': prior to intervention;'T 3': 3-month follow-up.*	*Improvements in SCAS (d=-0.50*) and CFSEQ (d=0.58*) from T1 to T3 for whole sample; not between T2 and T3 (across intervention). Improvements on both measures (d=-1.26*; d=-1.27*) for 'high risk' group between T2-T3.*

For peer review only - http://bmjopen.bmj.com/site/about/guidelines.xhtml

Stallard et al., 2014 [34] *(75%)*	*Cluster randomised controlled trial randomised through computer tool.* *Health-led FRIENDS (n=489) x School led FRIENDS (n=472) x Controls (n=401).* [1]	• *RCADS 30 - child & parent.* • *Penn State Worry Questionnaire.* • *RSES.* • *Bully / victim questionnaire.* • *Subjective wellbeing assessment.* • *SDQ- Parent version; teachers completed 'Impact scale'.*	*Baseline;6 months f-up; 12 months follow-up.*	*Improvement on total RCADS (d=0.20*) and social (d=-0.09*) & general anxiety subscales (d=-0.20*) - not depression. Smaller effect sizes in school led group (d=0.02*; d=0.11*; d=0.01*;). No statistical improvements on secondary outcome measures or teacher / parent rating scales.*

[1] Study sufficiently powered to detect change. [2] Power calculation provided but proportion lost to follow up (>15%) reduced sample required for adequate power. *Significant at p<0.5 level.

SCAS = Spence Children's Anxiety Scale; SDQ – Strength and Difficulties Questionnaire; PATHS = Promoting Alternative Thinking Strategies; PTRS = Paths teacher rating scale; T-POT = Teacher Pupil Observation Tool; CFSEQ = Culture-free Self-esteem questionnaire; RCADS = Revised Child Anxiety and Depression Scale; CSI = Coping Strategy Indicator

For peer review only

Table 3. Design and outcome characteristics of Secondary school-based studies.

Study (% quality rating)	Study Design	Measures	Follow-up	Effects / outcomes
**Boniwell et al. 2016 [35]** (37.5%)	Non-randomised control group pre-post design. 'Personal Wellbeing' intervention group (n=211) x control group (n=85).	• SLSS. • MSLSS. • PNASC. • Qualitative interviews.	Baseline; Post-intervention (10 month f-up)	No significant improvement on SLSS or MSLSS. Decrease in 'satisfaction with school'(d=0.4*) and 'friends' (d=-0.17) scores for whole sample. Decrease in positive affect for both intervention and control groups (d=-0.24*; -0.79*); increase in negative affect (d=0.54*) for control group. NB: Those lost to follow up (n=103) not accounted for in analysis.
**Challen et al. 2014 [36]** (78.1%)	Non-randomised pragmatic controlled trial. UKRP intervention (n=1016) group x Control (n=1894) group.[1]	• CDI. • RCMAS. • SDQ.	Baseline; post intervention (4-9 months); 1 yr follow-up; 2 year follow-up.	Small significant impact on CDI post-intervention (d=0.093*); not maintained at 1 or 2 years follow up. No significant effects on RCMAS or SDQ scores.
**Chisholm et al. 2016 [37]** (75%)	Pragmatic cluster randomised controlled trail, randomised by independent researcher. 'Contact and MH Education' (n=354) group x MH Education (n=303) group.[2] No 'as usual' controls.	• RIBS (not validated for adolescents). • MAKS (not validated for adolescents). • SDQ. • Resilience scale. • Helpseeking Q. • Focus groups.	Baseline - 2 weeks prior to intervention day; 2 weeks post-intervention day.	Statistical sig. improvements on several scales post intervention day for both groups - 'contact and education' and 'education only': Attitudinal based stigma (d=0.23*; d=0.25*), knowledge based stigma (d=0.54*; d=0.59*), mental health literacy (d=0.05; d=0.13*;) emotional wellbeing (d=0.16*; d=0.14*), and resilience (d=0.07; d=0.22*). No change in 'helpseeking'.

[1] Study sufficiently powered to detect change.
[2] Power calculation provided but proportion lost to follow up (>15%) reduced sample required for adequate power. *statistically significant at p < 0.05 level.
SLSS – Student's Life Satisfaction Scale; MSLSS = Multidimensional Students Life Satisfactions Scale; PNASC = Positive and Negative Affect Schedule for Children; CDI = Children's Depression Inventory; RCMAS = Revised Children's Manifest Anxiety Scale; SDQ = Strength and Difficulties Questionnaires; RIBS = Reported and Intended Behaviour Scale; MAKS = Mental Health Knowledge Schedule; WEMWBS = Warwick Edinburgh Mental Wellbeing Scale;

For peer review only - http://bmjopen.bmj.com/site/about/guidelines.xhtml

**Kuyken et al. 2013 [38]** (59%)	Non-randomised controlled feasibility study. MiSP intervention group (n=256) x control (n=266).	• WEMWBS.<br>• PSS.<br>• CES-D.<br>• Mindfulness practice.	Baseline; Post-intervention (9 weeks); 3 month follow-up.	Lower depression scores post-intervention (d=-0.29*). Improvement on all measures at 3 month follow-up (WEMWBS: d=0.15*; PSS: d=-0.09*; CES-D: d=-0.24*). Mindfulness practice significantly associated with greater gains across all measures (unable to calculate E.S.).
**Rice et al. 2015 [39]** (50%)	Non-randomised longitudinal design with three intervention conditions.<br>TRY intervention group (n=50) x CBT group (n=53) x MBCT group (n=54) x PHSE controls (n=99).	• SMFQ.<br>• CGT to measure reward seeking.<br>• DASC and corresponding response time.<br>• SCEPT to measure overgeneral memory.	Baseline; 9 week follow-up.	Statistical sig. changes in reward seeking in TRY group (d=0.12*); no change after CBT or MBCT. No statistically significant decrease in SMFQ across groups compared to PHSE controls. When comparing treatment groups only, TRY showed statistical reduction in SMFQ when compared with MBCT and CBT (d=-0.8*); reward-seeking moderated reductions in SMFQs scores (d=1.62*).
**Naylor et al. 2009 [40]** (56.3%)	Non-randomised pre-post control group study. MH intervention group (n=175) x Control group (n-242).[1]	• Mental Health Questionnaire (unvalidated).<br>• SDQ.	Baseline (1 week before intervention); 6 months post-intervention.	Improvement in MHQ with regards to awareness of depression causes (d=0.21*) and bullying (d=0.31*). Changes in specific SDQ subscales: 'conduct' (d=0.22*) and 'prosocial' (d=0.11*) but not on total difficulties.
**Stallard et al. 2013** (81.3%)	Cluster randomised controlled trial, randomised by computer.<br>UK-RAP intervention group (n=1753) x Attention controls (n=1673) x PHSE controls (n=1604)[1]	• SMFQ.<br>• CATS.<br>• RSES.<br>• RCADS.<br>• School connectedness.<br>• Attachment Questionnaire.<br>• European Quality of Life-5	Screening - SMFQ only; Baseline; 6 months follow-up; 12 months follow-up.	No significant effect on SMFQ at 12 months follow-up. Some effect of intervention on bullying status at 12 months, and Cannabis use at 6mo and 12 months follow-up. Intervention less useful than usual PHSE or attention controls for panic; less useful than usual PHSE on CATS 'personal failure' and general anxiety. Signs of benefits and harm of intervention found, all reported to be small effect sizes (data unavailable to calculate effect size).

PSS = Perceived Stress Scale; CES-D = Center for Epidemiologic Studies Depression Scale; SMFQ = Short Mood and Feelings Questionnaire; CGT = Cambridge Gambling Task; DASC = Dysfunctional Attitudes Scale for Children; SCEPT = Sentence completion for Events from the Past (SCEPT); CATS = Children's Automatic Thoughts Scale; RSES = Rosenberg Self Esteem Scale.

For peer review only - http://bmjopen.bmj.com/site/about/guidelines.xhtml

dimensions.	

[1] Study sufficiently powered to detect change. [2] Power calculation provided but proportion lost to follow up (>15%) reduced sample required for adequate power. *Significant at p<0.5 level.

peer review only

Implementation Issues

Common issues relating to implementation were found across all studies.

Fidelity

Fidelity to intervention delivery was highlighted as an issue in terms of both measurement and outcome. Studies used self-rated fidelity methods [32], external fidelity ratings on a proportion of sessions [31,34,36,37,41] or no fidelity rating methods reported at all. Studies commented variably on the possible effect of fidelity and 'treatment dosage' on outcomes. In Stallard et al.'s [37] study the health-led condition with 100% fidelity (i.e. administered all pieces of homework and activity tasks), was associated with significantly better outcomes than the school-led group who achieved 60-80% fidelity. 'High quality' workshops were also found to be related to greater declines in CDI measures [36]. Conversely, Berry et al. [31] found that fidelity (when applying an arbitrary '80%' rate of 'high' fidelity) was not found to be related to outcome.

Attrition

Investment from schools was raised as an issue as demonstrated by school participation and attrition [31,41] and failure to administer follow up measures as per study procedures [32,35]. All studies, with the exception of Stallard et al. [41] provided little information about school or participant characteristics of those who dropped out. This confounding factor may have positively biased results. For instance, in Kuyken et al's [38] study, teachers who delivered the mindfulness intervention had been invested in the intervention for approximately 2 years before the beginning of the study and attended regular supervision, demonstrating good motivation throughout the study which found positive outcomes.

Costs

Two studies actively explored health economic costs involved [31,41]. Cost-effectiveness was not calculated by Berry et al. [31] due to lack of impact, and Stallard et al. [41] concluded that the intervention was not cost-effective. Of note, both studies may have sustained high costs due to employing external facilitators to lead the intervention rather than teachers [41] and hiring 'coach consultants' to monitor delivery [31].

For peer review only - http://bmjopen.bmj.com/site/about/guidelines.xhtml

DISCUSSION

This review aimed to explore the effectiveness and study quality of universally delivered school-based interventions within the UK which aim to promote mental and emotional wellbeing, or prevent mental ill health. Several clear conclusions can be drawn from this review, while other issues require further clarity from future research.

How effective are universal school-based interventions in the UK that promote mental health, emotional wellbeing, or psychological resilience?

Based on the studies included in this review, the effectiveness of universal school based interventions remains mixed, and at best, modest. Where there were several positive outcomes, effect sizes were small and methodological issues rendered many results to be interpreted with caution.

Studies based in Primary schools seemed to find more encouraging results from CBT-based interventions on measures of anxiety, although most studies had methodological limitations relating to use of appropriate controls, and failure to include those lost to follow up in analysis. Positive results tended to fall in the older age range of Primary school pupils (9-12 years old).

Within the Secondary school population, the most positive results were obtained when delivering mental health education sessions, behavioural or mindfulness interventions. Two high powered, good quality studies evaluating CBT based interventions within secondary populations found few significant results and one study indicated possible detrimental impacts of the intervention compared to controls, although any effect sizes related to these findings were small.

It is curious that studies fail to detect promising effects in the older, secondary school, population. It could be argued that a 2 year follow-up is not sufficient to truly detect change or prevention during the developmentally-sensitive time that is adolescence. Arguably, the demands placed on adolescents merely change in nature rather than impact over time. Adolescent psychosocial development [42] is particularly vulnerable as individuals are required to manage academic demands as they progress through their school career, navigate

For peer review only - http://bmjopen.bmj.com/site/about/guidelines.xhtml

friendships, seek to develop self-identities and deal with the physiological changes that occur as they transition through puberty. It could be that the existence of such pervasive and fluctuating stressors juxtaposed with measurement issues, discussed below, contribute to the failure to detect significant results in secondary school populations. Or, that such interventions simply have less impact for this population.

What methodologies are being applied in UK schools when trialling interventions and what is the quality of these studies?

Methodological issues were predominant in this review. Only four of the studies were of 'excellent' quality and findings indicated a trend towards higher quality papers finding fewer positive results. Studies were weakened largely due to their lack of randomisation and blinding of researchers, and small sample sizes which likely rendered them underpowered to detect true effects.

While it was encouraging that initial consenting rates were high and remained reasonable throughout, study quality would benefit from better reporting of those lost to follow up who, possibly, could be a population of particular interest when considering the objective of promoting mental and emotional wellbeing for all within the school setting. Further, statistical methods used to account for such missing data require careful consideration to ensure that more stringent and conservative methods - for example, intent-to-treat analyses - are applied in school-based research. Otherwise, studies that instead apply a 'defined completers' or 'completers' analysis expose themselves to the risk of yielding false positives.

Another issue was the use of controls. Few studies explicitly provided details of the content controls groups received. Some indicated that controls may have already received materials available in the school around social and emotional wellbeing, which could reasonably have confounded results. Additionally, considering the demographic data provided, it is unlikely that the included studies accurately represent the cultural diversity of schools across the

For peer review only - http://bmjopen.bmj.com/site/about/guidelines.xhtml

UK, therefore caution should be taken when considering the generalizability of results.

The last prominent issue highlighted in this study was the diverse use of measures and length of follow up across studies, making it difficult to ascertain a coherent picture of measurement and effects in the current research base.

As commented in one study [36] and further afield [21], measurement issues within universal populations are particularly problematic due to common floor effects, particularly when using measures pertaining to the existence of mental health conditions. As has been well-documented, demonstrating improvement in 'high risk' groups is somewhat easier as baseline scores are often elevated providing scope for reduction [41]. Demonstrating change within a universal population is therefore inherently more difficult and requires careful thought when moving forward. Is it sufficient that the absence of a mental health condition equates to greater wellbeing or resilience as suggested by Boniwell et al. [35], or should researchers direct attention to explicitly measuring wellbeing and resilience and mechanisms of change within such constructs in order to truly operationalise factors relating to the prevention of mental health difficulties?

Few studies in this review used wellbeing or resilience measures. However, those that did [37, 38] found positive effects. While any meaning of these results must be taken with caution due to methodological issues, this nevertheless suggests that such measures are at least able to detect change within a universal population.

Only one study explored mechanisms of change [39] by using cognitive reasoning tests when comparing several interventions, and found that a behavioural intervention led to more reward-seeking and a reduction in mood symptoms. It would be of value to explore this further given the neurodevelopmental stage of early adolescence when frontal lobes are still maturing and neuronal connections continue to grow [43]. Consequently, the adolescent's ability to plan, problem solve and manipulate abstract information, as is arguably necessary in cognitive-based interventions, may be overridden by more disinhibited, emotionally driven impulses and the seeking of concrete rewards, as

For peer review only - http://bmjopen.bmj.com/site/about/guidelines.xhtml

may be seen in earlier adolescence and would potentially explain increased receptiveness to a behavioural rather than cognitive intervention.

What are the identified barriers in delivering and evaluating universal school based interventions?

Implementation barriers relating to fidelity to intervention delivery and costs were also raised within this review. Variance in fidelity measurement to confirm reliable manualised delivery was a recurring issue, which is of particular salience when delivery has been consistently argued to be related to outcome [10,12]. Intervention delivery itself varied between studies where school staff or external researchers delivered the courses. While results were mixed when comparing the effectiveness of teacher-led versus externally-led interventions, overall within this review the results were neutral suggesting, at best, that there is no negative impact of teacher delivery. While issues relating to treatment fidelity may be more prominent with teacher delivery, considering sustainability, it could be argued that this would be the optimal approach in school settings, especially considering the financial costs involved in employing external facilitators as demonstrated by two studies in this review [31, 41]. Further, research has indicated that pupils prefer both that mental health education be delivered by someone with a thorough knowledge of the subject, and for it to be delivered by someone they know e.g., a teacher [44].

No study in this review explored the impact on any allied services such as CAMHS. For instance, it may be useful to audit local CAMHS referral-rates whilst reviewing the effectiveness of school-based interventions, and whether an increase or decrease in referrals would be observed. Considering the absence of reliable positive outcomes at the individual level at this point, a systemic perspective could be of value when considering any cost benefits to the wider health and social care services.

Further, it was unclear from the review what local or national political or strategic drivers instigated each study, and indeed, the extent to which children and young people were consulted in the process, design and delivery of the

For peer review only - http://bmjopen.bmj.com/site/about/guidelines.xhtml

interventions. It was outside the scope of this review to explore the qualitative findings from the few studies that employed focus groups. Therefore, it is recommended that future qualitative reviews of school-based research are conducted in order to ensure that children's and young people's views as stakeholders in this work are sufficiently represented.

Limitations

This study was limited in its ability to source evaluations representative of the entire UK as the majority of studies were based in England. While efforts were made to source evaluations from elsewhere in the UK, the lack of validated measures or application of pre-post methodology meant that such evaluations from the 'grey literature' could not be included in this review. It should therefore be noted that there is much relevant work being conducted in schools across the UK. However, schools and local authorities should be urged to reliably evaluate their valuable efforts and contribute to the published literature, thereby demonstrating the important work being driven by teachers and policymakers nationwide.

This study was also limited in its date source in that only studies from the year 2000 were included in this review. While results from other systematic reviews suggested that little relevant research was done in the UK before this time, it could still be that some studies were missed due to this limit.

Implications

This review highlighted the need to employ robust methodological designs within school-based research in order for any effects to be interpreted meaningfully. Measurement issues exist where they do not adequately detect change in universal populations, and there is a wide variety of measures used ranging from 'clinical' to wellbeing measures. This review concludes that school-based researchers across the UK should attempt to come together to discuss ways to address this issue and improve coherence in the literature.

For peer review only - http://bmjopen.bmj.com/site/about/guidelines.xhtml

An additional, imperative implication from this review is the proactive inclusion and involvement of teachers in this work. As has been commented elsewhere [45] without the 'buy-in' from teachers, any school-based intervention is less likely to sustain or achieve positive outcomes. In a time of additional pressures on teachers, the need to feel in control of initiatives is key. Of note, two of the studies in this review included adult-focussed exercises for the teachers themselves as an adjunct to the intervention training. This approach may go further to assist teachers' stress management and understanding of mental health whilst attending to the needs of their pupils.

Conclusions

The current evidence suggests there are neutral to small effects of universal, school-based interventions in the UK that aim to promote emotional or mental wellbeing or prevention of mental health difficulties. Whilst the real-world limitations of conducting research in schools exists, robust, long-term methodologies need to be attempted when conducting research in this area in order to explore the longitudinal impact of school-based interventions on wellbeing. Academic attainment, school attendance and rates of high-risk presentations also need to be further explored. This requires adequate recording of fidelity, the use of validated measures sensitive to mechanisms of change, reporting of those lost to follow up and any adverse effects, and the use of qualitative data to supplement quantitative outcomes. Interventions in the existing UK-based literature include educational, behavioural, cognitive and mindfulness components, each demonstrating variable results. Nevertheless, national and local policy [25, 26, 27] indicates that there remains an appetite to develop work in this area in order to promote wellbeing outcomes for children and young people. In this case, further research collaborations are required across the UK to robustly demonstrate any benefits for pupils or on the wider system.

For peer review only - http://bmjopen.bmj.com/site/about/guidelines.xhtml

CONTRIBUTORSHIP

This piece of research was submitted in part fulfilment for a Doctorate degree in Clinical Psychology. KM (Dr Karen Mackenzie) was the main researcher and responsible for developing the research questions, conducting the search strategy and analysing results. CW (Professor Chris Williams) supervised this research and acted as co-rater during the search process. Many thanks to Dr Claire Adey (CA) who assisted in the quality rating process.

COMPETING INTERESTS

CW is President of BABCP- the lead body for CBT in the UK, and a CBT researcher and trainer. He is also author of a range of CBT-based resources including some aimed at primary and secondary school populations. These are available commercially as books, online courses, and classes. He receives royalty, and is shareholder and director of a company that commercialises these resources.

FUNDING STATEMENT

This research received no specific grant from any funding agency in the public, commercial or not-for-profit sectors.

DATA SHARING

Supplementary data available upon request to the author.

FIGURE LEGEND

Figure 1: PRISMA flow diagram

Table 1: Overview of interventions

Table 2: Study design and outcomes – Primary schools

Table 3: Study design and outcomes – Secondary schools

For peer review only

REFERENCES

1. Kieling C, Baker-Heningham H, Belfer M, et al. Child and adolescent mental health worldwide: evidence for action. *Lancet* 2011;378:1515–25.

2. Costello EJ, Mustillo S, Erkanli A, et al. Prevalence and development of psychiatric disorders in childhood and adolescence. *Arch Gen Psychiatry* 2003; 60:837–44.

3. Kendall, P. C., Safford, S., Flannery-Schroeder, E., & Webb, A. Child anxiety treatment: Outcomes in adolescence and impact on substance abuse and depression at 7.4 year follow-up. *Journal of Consulting and Clinical Psychology* 2004;72:276–287.

4. Collins, K. A., & Dozois, D. J. A. What are the active ingredients in preventative interventions for depression? *Clinical Psychology: Science and Practice* 2008;15:313–330.

5. Patel V, Flisher AJ, Hetrick S, McGorry P. Mental health of young people: a global public-health challenge. *The Lancet* 2007;369(9569):1302–13.

6. World Health Organization. Atlas: Child and Adolescent Mental Health Resources: Global Concerns, Implications for the Future. WHO, 2005.

7. Masia-Warner, C., Nangle, D. W., & Hansen, D. J. Bringing evidence-based child mental health services to the schools: General issues and specific populations. *Education and Treatment of Children* 2006;29:165–172.

8. Domitrovich CE, Bradshaw CP, Greenberg MT, Embry D, Poduska JM, Ialongo NS. Integrated models of school-based prevention: logic and theory. *Psychology in Schools*. 2010;47(1):71–88.

9. Vostanis, P., Humphrey, N., Fitzgerald, N., Deighton, J., & Wolpert, M. (2013). How do schools promote emotional wellbeing among their pupils? Findings from a national scoping survey of mental health provision in English schools. *Child and Adolescent Mental Health* 2013;18:151–157.

For peer review only - http://bmjopen.bmj.com/site/about/guidelines.xhtml

10. Durlak, JA, Weissberg, RP, Dymnicki, AB, Taylor, RD & Schellinger, KB. The Impact of Enhancing Students' Social and Emotional Learning: A Meta-Analysis of School-Based Universal Interventions. *Child Development* 2011;82(1):405–432.

11. Wells, J, Barlow, J & Stewart-Brown. A systematic review of universal approaches to mental health promotion in school. *Health Education* 2003;103(4): 197-220.

12. Neil, A.L. & Christensen, H. Efficacy and effectiveness of school-based prevention and early intervention programs for anxiety. *Clinical Psychology Review* 2009; 29(3):208–215.

13. Mychailyszyn. M.P., Brodman, D.M., Read, K.L., et al. Cognitive Behavioural School-based interventions for anxious and depressed youth: a meta-analysis of outcomes. *Clinical Psychology Science and Practice* 2012;19:129-153.

14. Weare, K & Nind, M. Mental health promotion and problem prevention in schools: what does the evidence say? *Health Promotion International* 2011;26(1): 29-69.

15. Merry, S., Hetrick, S., Cox, G.R., et al. Psychological and educational interventions for preventing depression in children and adolescents. *Cochrane Database of Systematic Reviews* 2011;12.DOI:10.1002/14651858.CD003380.pub3.

16. Corrieri, S., Heider, D., Conrad, I., et al. School-based prevention programs for depression and anxiety in adolescence: a systematic review. Health Promotion International 2013;29(3):427-441.

17. Spence, S.H., & Shortt, A.L. Research review: Can we justify widespread dissemination of universal based interventions for the preventions of depression among children and adolescents? *Journal of Child Psychology and Psychiatry* 2007;48(6):526-542.

18. Calear, al. & Christensen, H. Review of internet-based prevention and treatment programs for anxiety and depression in children and adolescents. *The Medical Journal of Australia* 2010;192(11):12–14.

For peer review only - http://bmjopen.bmj.com/site/about/guidelines.xhtml

19. Kavanagh, J., Oliver, S., Lorenc, T., et al. School based cognitive-behavioural interventions: A systematic review of effects and inequalities. *Health Sociology Review* 2009;18: 61-78.

20. Stallard, P. School-based intervention for depression and anxiety in children and adolescents. *Evidence-based Mental Health* 2013;16(3):60-61.

21. Reavley, N., Bassilos, B., Ryan, S., et al. Interventions to build resilience in Young People: a literature review. *Victorian Health Promotion Foundation* 2015. Melbourne.

22. Sancassiani, F., Pintus, E., Holte, A,. et al. Enhancing the Emotional and Social Skills of the Youth to promote their Wellbeing and Positive Development: A systematic review of Universal School-based Randomised Controlled Trials. C*linical Practice & Epidemiology in Mental Health* 2015;11:21-40.

23. National Institute of Clinical Excellence. *Promoting children's social and emotional well-being in primary education*. 2008. Retrieved from http://www.nice.org.uk/guidance/index. Accessed June 2016.

24. Cheney, G., Schlosser, A., Nash, P., et al. Targeted group-based interventions in schools to promote emotional well-being: A systematic review. *Clinical Child Psychology & Psychiatry* 2014;19(3):412–438

25. Department of Education. *Mental health and behaviour in schools: Departmental advice for school staff*. 2016. www.nationalarchives.gov.uk/doc/open-government-licence/version/3. Accessed June 2016.

26. Education Scotland (no publication date). Curriculum for Excellence. *Health and Wellbeing Across Learning: Responsibilities for All. Experience and Outcomes.* www.educationscotland.gov.uk/health_wellbeing_experiences_outcomes_tcm4-540031.pdf Accessed June 2016.

27. CYMRU. 'Thinking positively: Emotional health and well-being in schools and Early Years settings'. 2010 www.wales.gov.uk/educationandskills. Accessed June 2016.

For peer review only - http://bmjopen.bmj.com/site/about/guidelines.xhtml

28. Downs, S. H., & Black, N. The feasibility of creating a checklist for the assessment of the methodological quality both of randomized and non-randomized studies of health care interventions. *Journal of Epidemiological Community Health* 1998;52:377–384.

29. Popay, J., H. Roberts, A. Sowden, F et al. *Guidance on the Conduct of Narrative Synthesis in Systematic Reviews: Final Report*. Swindon: ESRC Methods Programme 2006.

30. Attwood, M., Meadows, S., Stallard, P., et al. Universal and targeted computerised cognitive behavioural therapy (Think, Feel, Do) for emotional health in schools: Results from two exploratory studies. *Child and Adolescent Mental Health* 2012;17(3):173-178.

31. Berry. F., Axford, N., Blower, S., et al. The effectiveness and micro-costing analysis of a Universal, School based, Social-Emotional Learning programme in the UK: A cluster-randomised controlled trial. *School Mental Health* 2016;8:238-256.

32. Collins, S., Woolfson, L. M ., & Durkin, K. Effects on coping skills and anxiety of a universal school-based mental health intervention delivered in Scottish primary schools. *School Psychology International* 2013; 35(1):85-100.

33. Stallard, P., Simpson, N., Anderson, S., et al. The FRIENDS Emotional Health Programme: Initial Findings from a School-Based Project. *Child & Adolescent Mental Health* 2007;12(1):32-37.

34. Stallard, P., Skyrabina, E., Taylor, G., et al. Classroom-based cognitive behaviour therapy (FRIENDS): A cluster randomised controlled trial to Prevent Anxiety in Children through Education in Schools (PACES). *The Lancet Psychiatry* 2014;1(3):185-192.

35. Boniwell. I., Osin, E.N., & Martinez, C. Teaching Happiness at school: non-randomised controlled mixed-methods feasibility study on the effectiveness of well-being lessons. *The Journal of Positive Psychology* 2016;11(1):85-98.

36. Challen, A. R., Machin. S.J., & Gillham J.E. The UK Resilience Programme: a school-based universal nonrandomized pragmatic controlled trial. *Journal of Consulting & Clinical Psychology* 2014;82(1):75-89.

For peer review only - http://bmjopen.bmj.com/site/about/guidelines.xhtml

37. Chisholm, K. E., Patteron, P., Torgerson, C., et al. Impact of contact on adolescents' mental health literacy and stigma: the SchoolSpace cluster randomised controlled trial. *BMC Open* 2016,6:e009435.

38. Kuyken, W., Weare, K., Obioha, C., et al. Effectiveness of the Mindfulness in Schools Programme: Non-randomised controlled feasibility study. *The British Journal of Psychiatry* 2013; 203(2):126-131

39. Rice, F., Rawal, A., Riglin, L., et al. Examining reward-seeking, negative self-beliefs and over-general autobiographical memory as mechanisms of change in classroom prevention programs for adolescent depression. *Journal of Affective Disorders* 2015;186:320-327.

40. Naylor, P., Cowie, H.E., Watters, S.J., et al. Impact of a mental health teaching programme on adolescents. *The British Journal of Psychiatry* 2009;194:365-370.

41. Stallard. P., Phillips. R., Montgomery, A.A., et al. A cluster RCT to determine the clinical effectiveness and cost-effectiveness of classroom-based cognitive-behavioural therapy (CBT) in reducing symptoms of depression in high-risk adolescents. *Health Technology Assessment* 2013;*17*(47).

42. Erikson, Erik H. Identity, Youth and Crisis. New York: Norton. 1968.

43. Spear, L.P. Adolescent Neurodevelopment. *Journal of Adolescent Health* 2013; 52(2):S7-13. doi:10.1016/j.jadohealth.2012.05.006

44. Woolfson, R, Woolfson, L, Mooney, L, Bryce, D. Young people's views of mental health education in secondary schools: A Scottish study. *Journal of Child: care, health and development* 2008;35(6):790-798.

45. Weare, K. Editorial: Child and adolescent mental health in schools. *Child and Adolescent Mental Health* 2015;20(2):6-8.

For peer review only - http://bmjopen.bmj.com/site/about/guidelines.xhtml

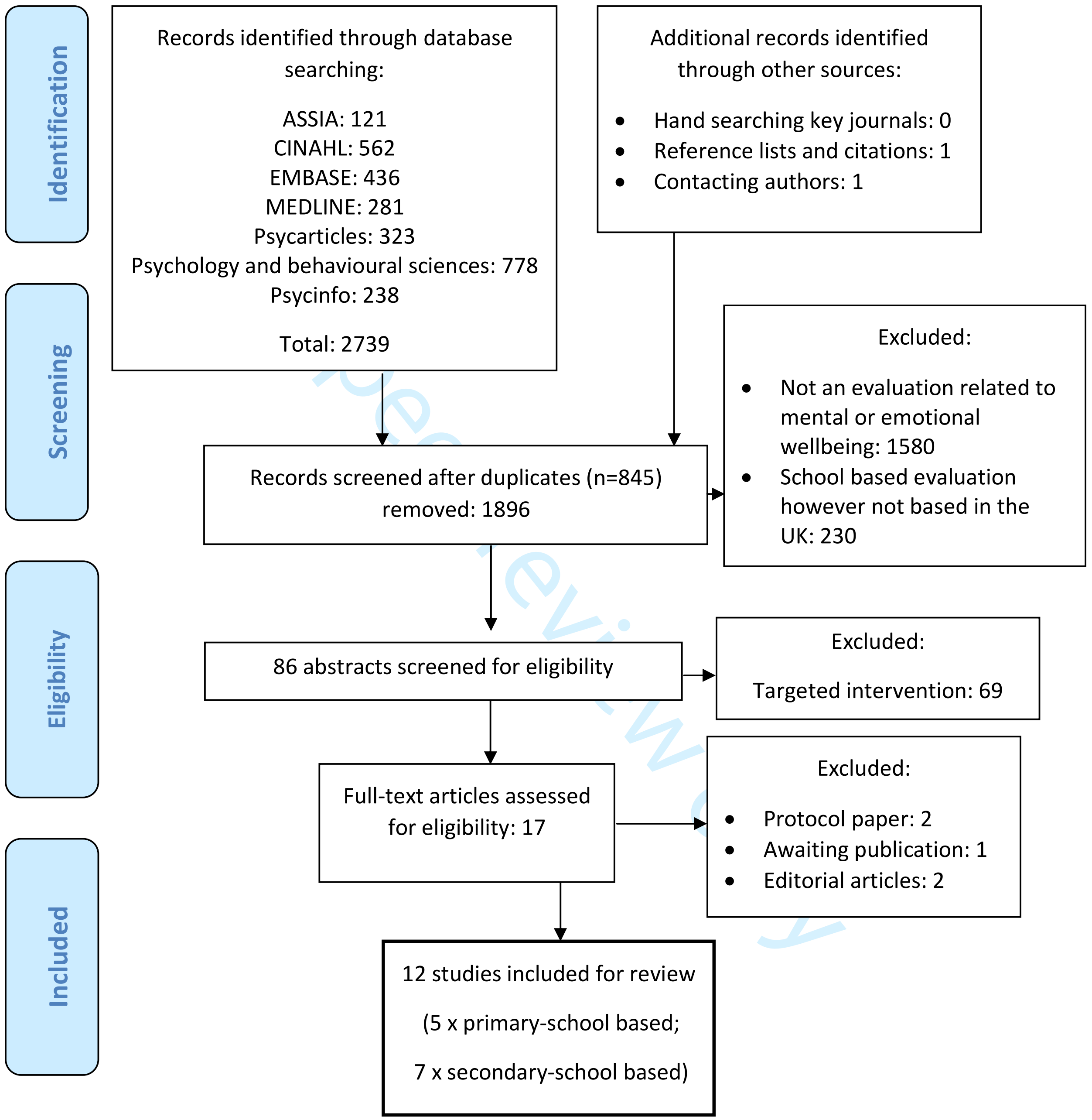

Figure 1: PRISMA diagram of systematic search process.

BMJ Open

Universal, school-based interventions to promote mental and emotional wellbeing. What is being done in the UK and does it work? A systematic review

Journal:	*BMJ Open*
Manuscript ID	bmjopen-2018-022560.R1
Article Type:	Research
Date Submitted by the Author:	12-Jun-2018
Complete List of Authors:	Mackenzie, Karen; NHS Ayrshire and Arran, Psychological Services<br>Williams, Christopher; University of Glasgow, Psychiatry
**Primary Subject Heading**:	Mental health
Secondary Subject Heading:	Public health
Keywords:	school based, intervention, wellbeing, review, resilience, MENTAL HEALTH

SCHOLARONE™
Manuscripts

For peer review only

Universal, school-based interventions to promote mental and emotional wellbeing. What is being done in the UK and does it work? A systematic review

Karen Mackenzie*, Chris Williams**

Address for correspondence:
*Psychological Services NHS Ayrshire and Arran
Ayrshire Central Hospital
Kilwinning Road
Irvine KA12 8SS
Email: karen.mackenzie@aapct.scot.nhs.uk

**Mental Health and Wellbeing
University of Glasgow
1st Floor, Administrative Building,
Gartnavel Royal Hospital
1055 Great Western Road
Glasgow
G12 0XY
E-mail: chris.williams@glasgow.ac.uk

Keywords: Review, school-based, resilience, wellbeing, intervention.

Word count: 5067 (excluding abstract, figures, tables and references).

For peer review only - http://bmjopen.bmj.com/site/about/guidelines.xhtml

ABSTRACT

Objectives: The present review aimed to assess the quality, content and evidence of efficacy of universally-delivered (to all pupils aged 5-16 years), school-based, mental health interventions designed to promote mental health/wellbeing and resilience, using a validated outcome measure and provided within the UK in order to inform UK schools-based wellbeing implementation.

Design: A systematic review of published literature set within UK mainstream school settings.

Data sources: Embase, Cinahl, Medline, PsycInfo, PsychArticles, ASSIA and Psychological and Behavioural Sciences published between 2000 and April 2016.

Eligibility criteria: Published in English; universal interventions that aimed to improve mental health/emotional wellbeing in a mainstream school environment; school pupils were the direct recipients of the intervention; pre-post design utilised allowing comparison using a validated outcome measure.

Data extraction and synthesis: 12 studies were identified including RCTs and non-controlled pre-post designs (5 primary school based, 7 secondary school based). A narrative synthesis was applied with study quality check (Downs and Black, 1998).

Results: Effectiveness of school-based universal interventions was found to be neutral or small with more positive effects found for poorer quality studies and those based in Primary schools (pupils aged 9-12 years old). Studies varied widely in their use of measures and study design. Only four studies were rated "excellent" quality. Methodological issues such as small sample size, varying course fidelity and lack of randomisation reduced overall study quality. Where there were several positive outcomes, effect sizes were small and methodological issues rendered many results to be interpreted with caution. Overall, results suggested a trend whereby higher quality studies reported less positive effects. The only study that conducted a health economic analysis suggested the intervention was not cost-effective.

Conclusions: The current evidence suggests there are neutral to small effects of universal, school-based interventions in the UK that aim to promote

For peer review only - http://bmjopen.bmj.com/site/about/guidelines.xhtml

emotional or mental wellbeing or the prevention of mental health difficulties. Robust, long-term methodologies need to be pursued ensuring adequate recording of fidelity, the use of validated measures sensitive to mechanisms of change, reporting of those lost to follow up and any adverse effects. Further high quality and large-scale research is required across the UK in order to robustly test any long-term benefits for pupils or on the wider educational or health system.

Limitations:

- Included papers largely based in England so unlikely to be representative of the cultural diversity within UK schools.
- Date limit excluded papers published prior to 2000.

Strengths:

- Addressed a gap in the literature.
- Used a robust methodology to review the literature in this area.
- Conclusions will help inform UK policy and practice as this topic continues to be debated in current health, education and political spheres.

INTRODUCTION

The mental and emotional wellbeing of children and young people has received increasing attention worldwide. It has been reported that the prevalence of mental health problems ranges from 10-20% [1], and that by the age of 18 up to 20% of young people will have experienced an emotional disorder [2]. Mental health conditions such as anxiety and depression often persist into adulthood [3] and have been associated with a range of negative outcomes including lower academic achievement, higher likelihood of health risk behaviours, self-harm and suicide [4, 5]. However, provision of services for those in need can be as low as 20% [6]. Such access issues to specialist services like CAMHS has meant that school-based interventions have been increasingly

For peer review only - http://bmjopen.bmj.com/site/about/guidelines.xhtml

explored due to their far reach [7] and existing infrastructure to support child development [8], while noting that schools need support to use the evidence base when applying such interventions [9].

Numerous systematic reviews and meta-analyses have been conducted to review the effectiveness of school-based, mental health interventions at both the universal (delivered to all pupils irrespective of perceived need), and targeted (delivered to vulnerable or 'high risk' individuals only) levels. Overall, this literature has indicated mixed results regarding efficacy of school-based interventions.

Findings have suggested positive effects on social emotional skills, self-concept, positive social behaviours, conduct problems, emotional distress and problem solving when reviewing school based, universal programs aiming to enhance social and emotional skills [10-11]. Further reviews found Cognitive Behavioural Therapy (CBT) formed the basis of the majority of anxiety prevention programs (78%) and over 75% of trials reported a significant reduction in anxiety [12]. CBT-based interventions were also tentatively endorsed as mildly effective in reducing depression (E.S. = 0.29) and moderately effective (E.S. = 0.50) in reducing anxiety symptoms [13].

With regards to optimal implementation, it has been noted that more positive outcomes were obtained for programs adopting a 'whole-school' approach, that lasted more than one year, and aimed to promote mental health rather than prevent mental illness [11]. A balance of both universal and targeted approaches has been recommended, along with accurate implementation of interventions [14].

However, the long-term impact and target audience of such initiatives has been questioned. A meta-analysis reviewing prevention of depression programmes found that while there was evidence of immediate post-intervention effects, these did not sustain over time (24-36 months) [15]. Moreover, a review evaluating both anxiety and depression programmes found that while the majority were effective for depression (65%) and anxiety (73%), the effect sizes were small (0.12 - 0.29) [16].

For peer review only - http://bmjopen.bmj.com/site/about/guidelines.xhtml

It has also been argued that universal prevention interventions are, overall, not efficacious [17-18], with targeted programs being most effective (E.S. = 0.21 to 1.40). Likewise, that while school-based CBT programmes have been demonstrated to lead to a short-term reduction in depression symptoms, interventions are most effective for those in the clinical range [19].

The literature has, therefore, conveyed conflicting results regarding the efficacy of universal school-based interventions whilst consistently in highlighting methodological issues within the existing research base. Common issues include a lack of active intervention controls [20], studies' operationalisation and measurements of 'resilience' lack homogeneity [21]; that weak program fidelity and treatment dosage impacts outcomes [10] and that there is insufficient use of validated, standardised measures and long-term follow up [22].

It is also noteworthy that the majority of reviews have focussed worldwide, with most reviewed interventions based in Australia, the US or Canada. No reviews have to date focused on studies in schools in the UK. This trend was also referenced in a NICE funded review [23] of targeted and universal school-based interventions who noted that though findings from international based research are helpful, the generalisability to the UK educational system is questionable. Education system differences between countries and continents such as funding, political drivers, curriculum pressures and workforce planning issues give rise to a need for reviews specifically within the UK context, especially while local funders and UK commissioners face calls to address rising mental health problems in schools. Therefore, it is particularly timely to have access to the most relevant information drawn from the current literature as it pertains to the UK educational system specifically.

One systematic review of targeted school-based interventions within the UK research has been conducted [24]. This found that nurture groups demonstrate an immediate positive impact on the social and emotional

For peer review only - http://bmjopen.bmj.com/site/about/guidelines.xhtml

wellbeing on vulnerable young people, however, that results from longer term follow-up studies are less clear.

The need to carry out a review of universal school-based interventions specifically within the UK context therefore remains. This is especially pertinent in light of the increasing emphasis from national government on developing CAMHS services within the UK, and the impetus on health and education services to work together in order to improve wellbeing outcomes for children and young people [25, 26, 27].

Review aims

The present review aims to fill this gap in the literature by focusing on universally-delivered, school-based mental health interventions provided within the UK only. The following questions will be explored:

1. *How effective are universal school-based interventions in the UK that promote mental health, emotional wellbeing, or psychological resilience and what tools are being used to measure effectiveness?*
2. *What methodologies are being applied in UK schools when trialling interventions and what is the quality of these studies?*
3. *What are the intervention characteristics e.g. delivery, content, target audience?*
4. *What are the identified barriers in delivering and evaluating universal school based interventions?*

Search Strategy

Electronic databases were searched for relevant published research on 14th April 2016: EMBASE, CINAHL, MEDLINE, PsycINFO, PsycArticles, ASSIA and Psychological and Behavioural Sciences. Selected journals relevant to the area were hand-searched (British Journal of Educational Psychology; British Journal of School Nursing). Previous reviews and relevant papers were reviewed and following consultation with University librarians, keyword search terms were identified and linked with the Boolean operators 'AND' and 'OR' (see supplementary file for search strategy examples):

For peer review only - http://bmjopen.bmj.com/site/about/guidelines.xhtml

Study design criteria were wide to allow for the diverse range of methodologies used to overcome challenges in school-based research. Search terms were, therefore, chosen primarily to promote sensitivity to the subject area. A limit date was set from 2000 to April 2016. The early date limit was selected as this area has been promoted by UK governmental policy largely within the last decade. Further, detailed appraisal of the previous systematic reviews in this area found few, if any, discovered studies prior to this date.

Study selection

The inclusion criteria were as follows:

- The intervention was based in a mainstream school environment;
- The intervention was universal in its application (i.e. to all pupils irrespective of need)
- Pupils were the direct recipients of the interventions;
- The study adopted a pre-post design;
- The intervention aimed to target mental health and/or emotional wellbeing;
- The study used a validated measure to quantitatively evaluate emotional or mental wellbeing outcomes and reported those outcomes;
- The study was published in English between 2000 and April 2016 in a peer reviewed journal.

Exclusion criteria included:

- The study aims or methodology did not fit the inclusion criteria.
- Any studies using a non-validated outcome measure as their primary outcome e.g. Likert scales that were unvalidated.
- Any studies using a purely qualitative methodology.

Details of included and excluded studies

Duplicate papers were excluded. Titles were screened to identify only those that clearly met inclusion criteria. Abstracts were assessed independently by the authors. Raters met to compare included papers. Where eligibility was unclear based on the abstract, full articles were retrieved and assessed jointly by raters. Reference lists of included papers were searched as well as previous

For peer review only - http://bmjopen.bmj.com/site/about/guidelines.xhtml

For peer review only

reviews on related topics. Articles citing included articles were also reviewed and one paper was sourced via this method. Authors of protocol papers were contacted leading to an additional paper being sourced. Experts in the field in Scotland, England, Northern Ireland and Wales were contacted regarding any other studies. However, none were eligible for inclusion. Twelve papers were included in the final review (see Figure 1).

(Editors: See attached Image)

Quality rating of studies

The Downs and Black [28] checklist was used to assess quality. This checklist assesses internal and external validity, selection bias and study power over 27 items. This checklist was used due to its utility in assessing studies relating to public health and its applicability to assess quality in both randomised and non-randomised studies. Reliability and validity assessment has found the Quality Index to have high internal consistency, good test-retest ($r = 0.88$) and inter-rater ($r = 0.75$) reliability and good face and criterion validity (0.90) [28].

A sample of papers were assessed by an independent researcher (CA). Any rating discrepancies were discussed and a shared decision reached. A decision was taken not to exclude any studies found to be of poor quality as the aim of this current review was to critique universal school-based interventions whilst acknowledging that the real-world implementation of such evaluations can be challenging and, as a result, may reasonably impact study quality.

Data extraction

Due to the heterogeneity of the studies, meta-analysis was not appropriate. A narrative synthesis was applied to explain the findings of this review in line with current guidance [29]. Information gathered from the studies included: study aim, intervention (model, duration, delivery), sample characteristics, study procedures, outcomes and measures, and results. Issues relating to the implementation, as well as effectiveness, of interventions were also noted from those studies commenting on such barriers.

Patient and Public Involvement

For peer review only - http://bmjopen.bmj.com/site/about/guidelines.xhtml

No patients or members of the public were directly involved in this piece of research.

RESULTS

Overview of interventions

Of the twelve studies sourced, five took place in primary schools [30-34] and seven took place in secondary schools [35-41]. An overview of study interventions based in Primary and Secondary schools can be found in Table 1.

Primary school studies:

The five studies within primary school settings evaluated interventions based on computerised CBT [30]; a teacher led intervention embedded within the curriculum (e.g. PATHS – 'Promoting Alternative Thinking Strategies' [31]); manualised anxiety interventions (e.g. a locally developed anxiety intervention, or the Australian developed 'FRIENDS' programme) delivered by both school staff (teachers and nurses) and external health staff (e.g. psychologists) [32-34].

Secondary school studies:

Three of the secondary school-based studies trialled interventions based on Cognitive Behavioural Therapy principles (e.g. UK Resilience programme (UKRP), Resourceful Adolescent Programme (RAP-UK) [36, 39, 41]) delivered by school staff [36], educational psychologists [39] and external facilitators [41]. Interventions were also said to include principles of Interpersonal Therapy (RAP-UK [41]) and behavioural approaches ('TRY' [39]).

One study trialled an intervention based on Positive Psychology [35], two studies trialled a mindfulness-based intervention [38-39] and two trialled locally developed mental health education sessions delivered to all pupils [37,40]. These interventions were led by trained school teachers [35, 38, 40], and trained volunteers [37]. All delivered the intervention during Personal Health and Social Education (PHSE) classes.

Methodological quality

The quality of studies ranged from 'poor' (34% [30]; 37.5% [35]) to 'excellent' (75% [34,37]; 78.1% [36]; 81.3% [41]).

For peer review only - http://bmjopen.bmj.com/site/about/guidelines.xhtml

Six studies used a randomised controlled pre-post design [30-32, 34, 37, 41]. The remaining were non-randomised pre-post designs and only one did not have a control group [33]. Some studies were particularly weak on their description of sample characteristics and representation of the population [30,35], reporting of those lost to follow up and accounting for those in the analysis [32,35], and the exploring of adverse events, of which only one study provided information [41]. Only six studies provided a power calculation [31, 34,36,37,40,41], most of which had samples sufficiently powered to determine an effect (except [37]). The remaining studies did not provide such information.

Of the eleven studies employing controls, six used controls from the same school in which the intervention was taking place (32,34,36,37,39). All other studies recruited controls from different schools.

Sample sizes ranged from 13 [30] to 5075 [31]. The age of participants ranged from 4 [31] to 16 years old [38,41] with the majority of studies targeting the early adolescent age range (9-12 years old) at the end of Primary school or at the beginning of Junior / Secondary school [30, 32, 34-37].

Table 1. Overview of interventions based in Primary and Secondary schools.

PRIMARY SCHOOLS

Study (Location)	*Sample*	*Study aim / hypothesis*	*Intervention - Theoretical model and Content*	*Intervention - Setting, Structure and Delivery*
Attwood et al., 2012 [30] ***(Bristol, England)***	*10-12 year old boys from two co-educational schools.* *(n=13)*	*A proof of concept study to explore the viability and possible benefits of a cCBT programme.*	*'Think, Feel, Do' - Based on CBT principles with a psychoeducation component. Cartoon characters guide users through various activities including: emotional recognition; linking thoughts, feelings and behaviours; identifying and challenging negative thoughts; and problem solving. Involves quizzes, practical exercises, videos, music and animation.*	*Six x 45min sessions delivered via an interactive multimedia CD-ROM.* *Took place within the school, facilitated by the researcher.*
Berry et al., 2016	*4-6 year old pupils*	*Test the effectiveness and cost-*	*'PATHS' (Promoting alternative thinking strategies). Aims to improve skills in five areas: self-*	*44 lessons in Year 1; 47 x lessons in Year 2. Delivered*

For peer review only - http://bmjopen.bmj.com/site/about/guidelines.xhtml

[31] ***(Birming-ham, England)***	*(n=5075; 56 x schools)*	*effectiveness of the intervention to reduce children's level of behavioural and emotional difficulty.*	*awareness, managing feelings, motivation, empathy and social skills. Lessons are developmentally sequenced and focus on techniques for self-control; emotional and interpersonal understanding steps for solving interpersonal problems; positive self-esteem and improved peer relationships.*	*by trained teachers within classroom.* *Manual provides teacher scripts, pictures, activity sheets, photos, posters, and home activities.*
Collins et al., 2013 [32] ***(South Lanark-shire, Scotland)***	*9-10 year old pupils (n=317; 9 schools; 18 classes).*	*To explore if anxiety & coping showed improvement post-intervention, and test effects of delivery.*	*'Lessons for living: Think Well, Do Well'. CBT based intervention to develop coping skills. A series of skills practice using interactive teaching methods. Children are guided to recognise emotional symptoms, reduce avoidant coping strategies, and focus on proactive problems solving and support-seeking.*	*Ten lessons delivered by a psychologist (n=103) & teacher (n=79) during PSE. Teachers provided with intervention manual following one-day training.*
Stallard et al., 2007 [33] ***(Bath & N.E. Somerset, England)***	*9-10 year old pupils (n=106; 3 schools; 4 classes).*	*To evaluate an Australian-originated intervention in the UK; test delivery by school nurses.*	*'FRIENDS - Feelings, Relax, I can do it, Explore solutions, Now reward, Don't forget practice, Smile'. Based on CBT principles it teaches children practice skills to: identify their anxious feelings and learn to relax; identify unhelpful thoughts and replace them with helpful thoughts; face and overcome problems and challenges.*	*Ten sessions delivered by school nurses who attended 2-day training. Lessons comprise group work, workbooks, role play and games. Parents invited to pre-intervention session.*
Stallard et al., 2014 [34] ***(Bath, N.E. Somerset, Swindon, Wiltshire, England)***	*9-10 year old pupils (n=1448; 45 x schools)*	*To assess the effectiveness of FRIENDS delivered by both health and school professionals on anxiety prevention.*	*As above (Stallard et al., 2007)*	*Nine x 60 min lessons delivered to whole classes. Health-led group: two trained facilitators. Teacher-led group: led by class teacher. All attended 2-day training.*

For peer review only - http://bmjopen.bmj.com/site/about/guidelines.xhtml

SECONDARY SCHOOLS

Study (Location)	*Sample*	*Study aim / hypothesis*	*Intervention - Theoretical model and Content*	*Intervention - Setting, Structure & Delivery*
Boniwell et al., 2016 [35] ***(S.E. London, England)***	*11-12 year old pupils (n=296; 2 x Haber-dashers' Aske's Fed. of Schools)*	*To test the efficacy of a new school programme for the promotion of happiness and wellbeing skills.*	*'Personal Wellbeing Lesson Curriculum'. Covers the 'scientific basis of happiness' focusing specifically on two core aspects: positive emotions / experiences and positive relationships. Based on theoretical constructs from wellbeing research and positive psychology e.g. 'three good things', forgiveness letter, gratitude visit.*	*Eighteen bi-weekly 50 min scripted lessons delivered to 8 classes by 4 teachers who attended 5-day training. Provided with lesson plans, PowerPoints and handouts.*
Challen et al., 2014 [36] ***(Greater London, N.W. England & N.E. England)***	*11-12 year old pupils (n= 2844; 16 x schools; 3 x L.As)*	*To evaluate a UK version of Penn Resiliency Program (PRP). Hypothesised high completion rates and reduction of depression symptoms.*	*'UK Resiliency Program'. Aims to build resilience and promote realistic thinking and adaptive coping, based on Ellis's 'Activating event-belief consequences model'. Teaches cognitive behavioural and social problem-solving skills; encourages accurate appraisal of situations; and assertiveness, negotiation and relaxation skills.*	*An 18-hour program delivered within the timetable at the teacher's discretion. Delivered by school staff who attended 10-day training in the US.*
Chisholm et al.,2016 [37] ***(Birming-ham, England)***	*12-13 year old pupils (n=769; 6 x schools).*	*To test whether contact with an individual with MH diagnosis plus education is more effective in reducing stigma, improving MH literacy and promoting wellbeing than education alone.*	*'Schoolspace'. A 10-module MH intervention designed by study researchers covering topics such as stress, depression, psychosis, different ways of thinking and a drama workshop. The 'contact' group had an individual facilitating who was a MH service user and had a diagnosis (e.g. psychosis, BPD) - this was revealed halfway through the day.*	*A one-day intervention within the school led by NHS staff, trained volunteers and MH service users.*
Kuyken et al., 2013 [38] ***(England)***	*12-16 year old pupils (n= 522; 12 x*	*To investigate the acceptability of a mindfulness programme for teachers and*	*'Mindfulness in Schools Program' (MiSP). Involved learning to direct attention to immediate experience with open-minded curiosity and acceptance. Skills were learned*	*Nine weekly scripted lessons delivered as part of the curriculum, or at lunchtime by 7*

For peer review only - http://bmjopen.bmj.com/site/about/guidelines.xhtml

	schools).	*students; test efficacy of programme on MH and wellbeing.*	*through practice sessions and everyday application. Mindfulness practice used to work with mental states and everyday stressors to cultivate wellbeing and promote mental health.*	*teachers trained and approved to deliver the MiSP curriculum.*
Rice et al., 2015 [39] ***(S.E. England)***	*13-14 year old pupils (n=256; 3 x schools).*	*To compare three types of intervention which may prevent adolescent depression and explore cognitive mechanisms involved with each.*	*'TRY' (Thinking about Reward in Young People). Aimed to enhance reward processing through actively selecting activities to lift mood.* *CBT. Aimed to change negative thinking patterns by encouraging evaluation of thoughts.* *'MBCT' (Mindfulness Based Cognitive Therapy). Aimed to promote awareness and acceptance of thoughts and to develop regulation of attention through guided meditation.* *Psychoeducation regarding depression was provided to all groups.*	*Eight weekly manualised sessions of each intervention delivered within 50-minute PHSE lessons by Educational Psychologists who attended regular supervision.*
Naylor et al. 2009 [40] ***(Greater London, England)***	*14-15 year old pupils (n=416; 2 x schools).*	*To explore whether teaching adolescents about mental health would result in gains in knowledge and empathy.*	*Mental health lessons. Topics included: stress, learning disability, depression, suicide / self-harm, eating disorders, and bullying using methods such as discussion, role playing and internet searching.*	*Six 50 min weekly lessons delivered by 7 group tutors from pastoral care who attended a 1-day training from researchers.*
Stallard et al., 2013 [41] ***(Bath, N.E. Somerset, Bristol, Wiltshire, Notting-hamshire, England)***	*12-16 year old pupils (n=5030; 8 x schools, 28 x year groups).*	*To assess effects of classroom based CBT on symptoms of depression and in relation to other aspects of psychological wellbeing and specific demographic sub-groups.*	*'RAP-UK: Resourceful Adolescent Programme'. A depression prevention programme based on CBT and interpersonal therapy principles adapted to fit the UK curriculum. Key elements include: personal strengths, helpful thinking, keeping calm, problem solving, support networks and keeping the peace. Students complete workbooks as they progress.*	*Nine 50-60 min manualised lessons delivered within the PSHE curriculum by two trained facilitators external to the school.* *Two booster sessions offered to schools at 6 month follow up.*

For peer review only - http://bmjopen.bmj.com/site/about/guidelines.xhtml

EFFECTIVENESS OF INTERVENTIONS

An overview of study characteristics and outcomes can be found in Tables 2 and 3.

Data collection and measurement

Studies varied widely in their use of measures. Measures used to rate depressive symptoms included the Children's Depression Inventory (CDI) [36], the Short Mood and Feelings Questionnaire (SMFQ) [39, 41] and the Center for Epidemiological Studies – Depression Scale (CES-D) [38]. Measures used to rate anxiety included the Revised Children's Anxiety and Depression Scale (RCADS) [34, 41], Revised Children's Manifest Anxiety Scale (RCMAS) [36], Penn State Worry questionnaire [41] and the Spence anxiety scale [30,32,33]. Measures used to capture different methods of coping related to symptoms of anxiety or depression included: Children's Automatic Thoughts Scale (CATS) [41], Coping Strategy Indicator (CSI) [32], Sentence Completion for Events in the Past Test (SCEPT) [39], and Perceived Stress Scale (PSS) [38]. Two studies used measures related specifically to wellbeing or resilience: Warwick-Edinburgh Mental Wellbeing Scale (WEMWBS) [38] and the Resilience Scale [37] and others used measures related to self-esteem [33,34,41] and life satisfaction [35]. The Strength and Difficulties Questionnaire (SDQ) was the most commonly used measure said to rate behavioural, emotional difficulties and overall functioning and either the child, parent or teacher version was used in six of the twelve studies [30,31,33,36,37,40]. Studies varied according to the length of follow up ranging from 4 weeks [37], to 2 years [41]. Four of the twelve studies sought to obtain qualitative, as well as quantitative data [30,35,37,41]. However, it was beyond the scope of this paper to comment on qualitative findings.

Due to the heterogeneity of studies, the effectiveness of each intervention approach will be discussed in turn. Overall, results suggested a trend whereby higher quality studies reported less positive effects.

Studies trialling bespoke mental health education programmes (n=3; [40,37,35] – all in secondary schools).

Two studies found small (d=0.11-0.22) but significant improvements in total and subscale SDQ scores for those that received mental health education.

For peer review only - http://bmjopen.bmj.com/site/about/guidelines.xhtml

However, of those, it is noteworthy that Chisholm et al. [37] did not employ a non-intervention condition. Boniwell et al. [35] trialled a bespoke intervention based on Positive Psychology principles and found a decrease in outcomes of life satisfaction and an increase in negative affect for both groups. However, this was less so for the intervention group (d=-0.24 compared to d=-0.79) which was interpreted as the intervention having a 'buffering effect' at a time of stress for the pupils.

Studies trialling CBT-based interventions [n= 8; 30-34,36,39,41]. These are described by setting (Primary and then Secondary).

Primary schools

All primary-school based studies trialled interventions pertaining to altering thinking styles based on CBT principles. Four studies, three of which employed a control arm, reported statistically positive outcomes on anxiety-related measures following interventions including FRIENDS [33,34], 'Think Feel Do' [30] and locally developed CBT programmes [32] with larger effects for those in 'high risk' groups (d=-1.26; [33] – no control arm). Methodological issues such as a small sample size and significant group differences at baseline (n=13;[30]), failure to include those lost to follow-up in analysis [32], lack of controls [33], and small effect sizes for universal samples (d=0.01 - 0.2) [34] should be noted when taking inference from those results. Mixed results were found in relation to delivery, with stronger effects found in interventions led by health professionals (d=0.2) versus school staff (d=0.02) [34], or no difference between psychologist or teacher-led interventions [32]. A sufficiently powered, good-quality study evaluating the use of PATHS within the curriculum found few, small significant results (d = 0.06 – 0.14; teacher-rated intervention measure) at 12-month follow up, and no effects on any measure at 24-month follow up [31].

Secondary schools

Fewer significant outcomes were found in trials based within secondary school populations. Small (d=0.093), but short-lived positive outcomes were found on the CDI for those in the UKRP intervention [36]. Mixed results were found for those in the UK-RAP intervention, with results indicating some beneficial and also potentially negative outcomes [41] although all with small

For peer review only - http://bmjopen.bmj.com/site/about/guidelines.xhtml

effect sizes. Both were high quality, longitudinal, well-powered studies employing robust methodologies. Further, no effects were found in the CBT group when compared with as-usual controls or other treatments in a smaller study looking at mechanisms of change [39]. In the same study, a behavioural intervention (TRY) was found to have positive effects on reward-seeking behaviour and SMFQ measure (d=-0.8) when compared with other treatments; however, this finding was not confirmed when compared with PHSE-as-usual controls.

Studies using mindfulness-based interventions (n=2; [38,39] - both in secondary schools).

Positive outcomes were found in a feasibility study evaluating a mindfulness-based intervention [38] which yielded statistically significant, modest effects on both depression (CES-D: d=-0.24) and wellbeing (WEMWBS: d=0.15) measures. Due to small sample sizes this study was likely to be underpowered; however, outcomes were sustained at 3 months follow up and were associated with greater mindfulness practice. No significant outcomes were found in a smaller study trialling MBCT on measures of mood (SMFQ) or reward-seeking [39].

For peer review only - http://bmjopen.bmj.com/site/about/guidelines.xhtml

Table 2. Design and outcome characteristics of primary-school based studies.

Study (% quality rating)	Study Design	Measures	Follow-up	Effects / Outcomes
**Attwood et al., 2012 [30]** (34%)	Randomised pre-post intervention evaluation using opportunistic sample. No blinding or randomisation procedure reported. 'cCBT' (n=6) x control group (n=7).	• SCAS - Parent & Child version.<br>• SDQ - Parent version.<br>• Focus groups (n=8)	Baseline; 6-weeks post intervention.	Significant reduction in SCAS-C 'social' (d=0.49*) and 'general anxiety' (d=0.48*) subscales (NB: Intervention group significantly higher on SCAS at baseline). No effects on parent rated measures.
**Berry et al., 2016 [31]** (68.8%)	Randomised controlled trial; web randomisation system.<br><br>29 schools 'PATHS' intervention x 27 schools WL Control. [1]	• SDQ - teacher version.<br>• PATHS teacher rating scale (PTRS).<br>• T-POT.	Baseline; 12 month post-intervention; 24 month post intervention.	No differences on SDQ at 12-month F-up. Some significant results on subscales of PTRS at 12 month f-up (Social competence: d=0.09*; Aggression: d=0.14*; Inattention: d=-0.06*; Peer relations: -0.10*). Not maintained at 24 month follow up.
**Collins et al., 2013 [32]** (46.9%)	Randomised 3 x 3 mixed design. No randomisation procedure reported.<br><br>Psychologist led anxiety intervention (n=103) x Teacher led anxiety intervention (n=79) x Controls (n=135).	• CSI<br>• SCAS -Child version administered by teachers.	Baseline; post-intervention; (within 3 weeks of end); 6 months follow up.	Improvement in psychologist-led and teacher-led groups on SCAS-C (d=0.41*; d=0.31*) & CSI 'Avoidance' (d=0.31*; d=0.31*) and 'problem solving' (d=-0.66*; d=0.52*) subscales. No difference between psychologist or teacher-led groups. SCAS-C outcomes maintained at 6 months follow up (d=0.39*; d=0.39*). NB: Those lost to follow up (n=155) not included in analysis.
**Stallard et al., 2007 [33]** (43.4%)	Pre- post evaluation of pupils (n=106) from 3 schools taking part in the FRIENDS intervention.<br><br>No controls employed.	• SCAS-Child version.<br>• CFSEQ.	'T1': 6 month prior; 'T2': prior to intervention;'T 3': 3-month follow-up.	Improvements in SCAS (d=-0.50*) and CFSEQ (d=0.58*) from T1 to T3 for whole sample; not between T2 and T3 (across intervention). Improvements on both measures (d=-1.26*; d=-1.27*) for 'high risk' group between T2-T3.

For peer review only - http://bmjopen.bmj.com/site/about/guidelines.xhtml

Stallard et al., 2014 [34] *(75%)*	*Cluster randomised controlled trial randomised through computer tool.* *Health-led FRIENDS (n=489) x School led FRIENDS (n=472) x Controls (n=401).* [1]	• *RCADS 30 - child & parent.* • *Penn State Worry Questionnaire.* • *RSES.* • *Bully / victim questionnaire.* • *Subjective wellbeing assessment.* • *SDQ- Parent version; teachers completed 'Impact scale'.*	*Baseline;6 months f-up; 12 months follow-up.*	*Improvement on total RCADS (d=0.20*) and social (d=-0.09*) & general anxiety subscales (d=-0.20*) - not depression. Smaller effect sizes in school led group (d=0.02*; d=0.11*; d=0.01*;). No statistical improvements on secondary outcome measures or teacher / parent rating scales.*

[1] Study sufficiently powered to detect change. [2] Power calculation provided but proportion lost to follow up (>15%) reduced sample required for adequate power. *Significant at p<0.5 level.

SCAS = Spence Children's Anxiety Scale; SDQ – Strength and Difficulties Questionnaire; PATHS = Promoting Alternative Thinking Strategies; PTRS = Paths teacher rating scale; T-POT = Teacher Pupil Observation Tool; CFSEQ = Culture-free Self-esteem questionnaire; RCADS = Revised Child Anxiety and Depression Scale; CSI = Coping Strategy Indicator

For peer review only - http://bmjopen.bmj.com/site/about/guidelines.xhtml

Table 3. Design and outcome characteristics of Secondary school-based studies.

**Study** (% quality rating)	*Study Design*	*Measures*	*Follow-up*	*Effects / outcomes*
**Boniwell et al. 2016 [35]** (37.5%)	*Non-randomised control group pre-post design.* *'Personal Wellbeing' intervention group (n=211) x control group (n=85).*	• *SLSS.* • *MSLSS.* • *PNASC.* • *Qualitative interviews.*	*Baseline; Post-intervention (10 month f-up)*	*No significant improvement on SLSS or MSLSS. Decrease in 'satisfaction with school'(d=0.4*) and 'friends' (d=-0.17) scores for whole sample. Decrease in positive affect for both intervention and control groups (d=-0.24*; -0.79*); increase in negative affect (d=0.54*) for control group. NB: Those lost to follow up (n=103) not accounted for in analysis.*
**Challen et al. 2014 [36]** (78.1%)	*Non-randomised pragmatic controlled trial.* *UKRP intervention (n=1016) group x Control (n=1894) group.*[1]	• *CDI.* • *RCMAS.* • *SDQ.*	*Baseline; post intervention (4-9 months); 1 yr follow-up; 2 year follow-up.*	*Small significant impact on CDI post-intervention (d=0.093*); not maintained at 1 or 2 years follow up. No significant effects on RCMAS or SDQ scores.*
**Chisholm et al. 2016 [37]** (75%)	*Pragmatic cluster randomised controlled trail, randomised by independent researcher.* *'Contact and MH Education' (n=354) group x MH Education (n=303) group.*[2] *No 'as usual' controls.*	• *RIBS (not validated for adolescents).* • *MAKS (not validated for adolescents).* • *SDQ.* • *Resilience scale.* • *Helpseeking Q.* • *Focus groups.*	*Baseline - 2 weeks prior to intervention day;* *2 weeks post-intervention day.*	*Statistical sig. improvements on several scales post intervention day for both groups - 'contact and education' and 'education only': Attitudinal based stigma (d=0.23*; d=0.25*), knowledge based stigma (d=0.54*; d=0.59*), mental health literacy (d=0.05; d=0.13*;) emotional wellbeing (d=0.16*; d=0.14*), and resilience (d=0.07; d=0.22*). No change in 'helpseeking'.*

[1] Study sufficiently powered to detect change.
[2] Power calculation provided but proportion lost to follow up (>15%) reduced sample required for adequate power. *statistically significant at $p < 0.05$ level.
SLSS – Student's Life Satisfaction Scale; MSLSS = Multidimensional Students Life Satisfactions Scale; PNASC = Positive and Negative Affect Schedule for Children; CDI = Children's Depression Inventory; RCMAS = Revised Children's Manifest Anxiety Scale; SDQ = Strength and Difficulties Questionnaires; RIBS = Reported and Intended Behaviour Scale; MAKS = Mental Health Knowledge Schedule; WEMWBS = Warwick Edinburgh Mental Wellbeing Scale;

For peer review only - http://bmjopen.bmj.com/site/about/guidelines.xhtml

**Kuyken et al. 2013 [38]** (59%)	*Non-randomised controlled feasibility study. MiSP intervention group (n=256) x control (n=266).*	• *WEMWBS.*<br>• *PSS.*<br>• *CES-D.*<br>• *Mindfulness practice.*	*Baseline; Post-intervention (9 weeks); 3 month follow-up.*	*Lower depression scores post-intervention (d=-0.29*). Improvement on all measures at 3 month follow-up (WEMWBS: d=0.15*; PSS: d=-0.09*; CES-D: d=-0.24*). Mindfulness practice significantly associated with greater gains across all measures (unable to calculate E.S.).*
**Rice et al. 2015 [39]** (50%)	*Non-randomised longitudinal design with three intervention conditions.*<br>*TRY intervention group (n=50) x CBT group (n=53) x MBCT group (n=54) x PHSE controls (n=99).*	• *SMFQ.*<br>• *CGT to measure reward seeking.*<br>• *DASC and corresponding response time.*<br>• *SCEPT to measure overgeneral memory.*	*Baseline; 9 week follow-up.*	*Statistical sig. changes in reward seeking in TRY group (d=0.12*); no change after CBT or MBCT. No statistically significant decrease in SMFQ across groups compared to PHSE controls. When comparing treatment groups only, TRY showed statistical reduction in SMFQ when compared with MBCT and CBT (d=-0.8*); reward-seeking moderated reductions in SMFQs scores (d=1.62*).*
**Naylor et al. 2009 [40]** (56.3%)	*Non-randomised pre-post control group study. MH intervention group (n=175) x Control group (n-242).*[1]	• *Mental Health Questionnaire (unvalidated).*<br>• *SDQ.*	*Baseline (1 week before intervention); 6 months post-intervention.*	*Improvement in MHQ with regards to awareness of depression causes (d=0.21*) and bullying (d=0.31*). Changes in specific SDQ subscales: 'conduct' (d=0.22*) and 'prosocial' (d=0.11*) but not on total difficulties.*
**Stallard et al. 2013** (81.3%)	*Cluster randomised controlled trial, randomised by computer.*<br>*UK-RAP intervention group (n=1753) x Attention controls (n=1673) x PHSE controls (n=1604)*[1]	• *SMFQ.*<br>• *CATS.*<br>• *RSES.*<br>• *RCADS.*<br>• *School connectedness.*<br>• *Attachment Questionnaire.*<br>• *European Quality of Life-5 dimensions.*	*Screening - SMFQ only; Baseline; 6 months follow-up; 12 months follow-up.*	*No significant effect on SMFQ at 12 months follow-up. Some effect of intervention on bullying status at 12 months, and Cannabis use at 6mo and 12 months follow-up. Intervention less useful than usual PHSE or attention controls for panic; less useful than usual PHSE on CATS 'personal failure' and general anxiety. Signs of benefits and harm of intervention found, all reported to be small effect sizes (data unavailable to calculate effect size).*

PSS = Perceived Stress Scale; CES-D = Center for Epidemiologic Studies Depression Scale; SMFQ = Short Mood and Feelings Questionnaire; CGT = Cambridge Gambling Task; DASC = Dysfunctional Attitudes Scale for Children; SCEPT = Sentence completion for Events from the Past (SCEPT); CATS = Children's Automatic Thoughts Scale; RSES = Rosenberg Self Esteem Scale.

For peer review only - http://bmjopen.bmj.com/site/about/guidelines.xhtml

[1] Study sufficiently powered to detect change. [2] Power calculation provided but proportion lost to follow up (>15%) reduced sample required for adequate power. *Significant at p<0.5 level.

For peer review only

Implementation Issues

Common issues relating to implementation were found across all studies.

Fidelity

Fidelity to intervention delivery was highlighted as an issue in terms of both measurement and outcome. Studies used self-rated fidelity methods [32], external fidelity ratings on a proportion of sessions [31,34,36,37,41] or no fidelity rating methods reported at all. Studies commented variably on the possible effect of fidelity and 'treatment dosage' on outcomes. In Stallard et al.'s [37] study the health-led condition with 100% fidelity (i.e. administered all pieces of homework and activity tasks), was associated with significantly better outcomes than the school-led group who achieved 60-80% fidelity. 'High quality' workshops were also found to be related to greater declines in CDI measures [36]. Conversely, Berry et al. [31] found that fidelity (when applying an arbitrary '80%' rate of 'high' fidelity) was not found to be related to outcome.

Attrition

Investment from schools was raised as an issue as demonstrated by school participation and attrition [31,41] and failure to administer follow up measures as per study procedures [32,35]. All studies, with the exception of Stallard et al. [41] provided little information about school or participant characteristics of those who dropped out. This confounding factor may have positively biased results. For instance, in Kuyken et al's [38] study, teachers who delivered the mindfulness intervention had been invested in the intervention for approximately 2 years before the beginning of the study and attended regular supervision, demonstrating good motivation throughout the study which found positive outcomes.

Costs

Two studies actively explored health economic costs involved [31,41]. Cost-effectiveness was not calculated by Berry et al. [31] due to lack of impact, and Stallard et al. [41] concluded that the intervention was not cost-effective. Of note, both studies may have sustained high costs due to employing external facilitators to lead the intervention rather than teachers [41] and hiring 'coach consultants' to monitor delivery [31].

For peer review only - http://bmjopen.bmj.com/site/about/guidelines.xhtml

DISCUSSION

This review aimed to explore the effectiveness and study quality of universally delivered school-based interventions within the UK which aim to promote mental and emotional wellbeing, or prevent mental ill health. Several clear conclusions can be drawn from this review, while other issues require further clarity from future research.

How effective are universal school-based interventions in the UK that promote mental health, emotional wellbeing, or psychological resilience?

Based on the studies included in this review, the effectiveness of universal school based interventions remains mixed, and at best, modest. Where there were several positive outcomes, effect sizes were small and methodological issues rendered many results to be interpreted with caution. This prudent finding echoes the somewhat mixed results from worldwide reviews [10 – 23], where while several positive evaluations exist, this finding is not consistent when applied across diverse settings and populations, which calls into question the overall generalisability of school based interventions in the literature to real world environments.

Notwithstanding, this current review focussing on UK schools only found that studies based in Primary schools seemed to find more encouraging results from CBT-based interventions on measures of anxiety, although most studies had methodological limitations relating to use of appropriate controls, and failure to include those lost to follow up in analysis. Positive results tended to fall in the older age range of Primary school pupils (9-12 years old).

Within the Secondary school population, the most positive results were obtained when delivering mental health education sessions, behavioural or mindfulness interventions. Two high powered, good quality studies evaluating CBT based interventions within secondary populations found few significant results and one study indicated possible detrimental impacts of the intervention compared to controls, although any effect sizes related to these findings were small.

It is curious that studies fail to detect promising effects in the older, secondary school, population. It could be argued that a 2 year follow-up is not

For peer review only - http://bmjopen.bmj.com/site/about/guidelines.xhtml

sufficient to truly detect change or prevention during the developmentally-sensitive time that is adolescence. Arguably, the demands placed on adolescents merely change in nature rather than impact over time. Adolescent psychosocial development [42] is particularly vulnerable as individuals are required to manage academic demands as they progress through their school career, navigate friendships, seek to develop self-identities and deal with the physiological changes that occur as they transition through puberty. It could be that the existence of such pervasive and fluctuating stressors juxtaposed with measurement issues, discussed below, contribute to the failure to detect significant results in secondary school populations. Or, that such interventions simply have less impact for this population.

What methodologies are being applied in UK schools when trialling interventions and what is the quality of these studies?

Methodological issues were predominant in this review. Only four of the studies were of 'excellent' quality and findings indicated a trend towards higher quality papers finding fewer positive results. Studies were weakened largely due to their lack of randomisation and blinding of researchers, and small sample sizes which likely rendered them underpowered to detect true effects.

While it was encouraging that initial consenting rates were high and remained reasonable throughout, study quality would benefit from better reporting of those lost to follow up who, possibly, could be a population of particular interest when considering the objective of promoting mental and emotional wellbeing for all within the school setting. Further, statistical methods used to account for such missing data require careful consideration to ensure that more stringent and conservative methods - for example, intent-to-treat analyses - are applied in school-based research. Otherwise, studies that instead apply a 'defined completers' or 'completers' analysis expose themselves to the risk of yielding false positives.

Another issue was the use of controls. Few studies explicitly provided details of the content controls groups received. Some indicated that controls may

For peer review only - http://bmjopen.bmj.com/site/about/guidelines.xhtml

have already received materials available in the school around social and emotional wellbeing, which could reasonably have confounded results. Additionally, considering the demographic data provided, it is unlikely that the included studies accurately represent the cultural diversity of schools across the UK, therefore caution should be taken when considering the generalizability of results.

The last prominent issue highlighted in this study was the diverse use of measures and length of follow up across studies, making it difficult to ascertain a coherent picture of measurement and effects in the current research base.

As commented in one study [36] and further afield [21], measurement issues within universal populations are particularly problematic due to common floor effects, particularly when using measures pertaining to the existence of mental health conditions. As has been well-documented, demonstrating improvement in ‘high risk’ groups is somewhat easier as baseline scores are often elevated providing scope for reduction [41]. Demonstrating change within a universal population is therefore inherently more difficult and requires careful thought when moving forward. Is it sufficient that the absence of a mental health condition equates to greater wellbeing or resilience as suggested by Boniwell et al. [35], or should researchers direct attention to explicitly measuring wellbeing and resilience and mechanisms of change within such constructs in order to truly operationalise factors relating to the prevention of mental health difficulties?

Few studies in this review used wellbeing or resilience measures. However, those that did [37, 38] found positive effects. While any meaning of these results must be taken with caution due to methodological issues, this nevertheless suggests that such measures are at least able to detect change within a universal population.

Only one study explored mechanisms of change [39] by using cognitive reasoning tests when comparing several interventions, and found that a behavioural intervention led to more reward-seeking and a reduction in mood symptoms. It would be of value to explore this further given the neurodevelopmental stage of early adolescence when frontal lobes are still maturing and neuronal connections continue to grow [43]. Consequently, the

For peer review only - http://bmjopen.bmj.com/site/about/guidelines.xhtml

adolescent's ability to plan, problem solve and manipulate abstract information, as is arguably necessary in cognitive-based interventions, may be overridden by more disinhibited, emotionally driven impulses and the seeking of concrete rewards, as may be seen in earlier adolescence and would potentially explain increased receptiveness to a behavioural rather than cognitive intervention.

It could also be of value that future studies take a more holistic perspective of general wellbeing during evaluation of universal populations. Such indicators may include school attendance, exam completion, referrals rates to local CAMHS, academic outcomes, long-term mental and physical health outcomes, occupational or further education uptake, as well as important qualitative components.

What are the identified barriers in delivering and evaluating universal school based interventions?

Implementation barriers relating to fidelity to intervention delivery and costs were also raised within this review. Variance in fidelity measurement to confirm reliable manualised delivery was a recurring issue, which is of particular salience when delivery has been consistently argued to be related to outcome [10,12]. Intervention delivery itself varied between studies where school staff or external researchers delivered the courses. While results were mixed when comparing the effectiveness of teacher-led versus externally-led interventions, overall within this review the results were neutral suggesting, at best, that there is no negative impact of teacher delivery. While issues relating to treatment fidelity may be more prominent with teacher delivery, considering sustainability, it could be argued that this would be the optimal approach in school settings, especially considering the financial costs involved in employing external facilitators as demonstrated by two studies in this review [31, 41]. Further, research has indicated that pupils prefer both that mental health education be delivered by someone with a thorough knowledge of the subject, and for it to be delivered by someone they know e.g., a teacher [44].

No study in this review explored the impact on any allied services such as CAMHS. For instance, it may be useful to audit local CAMHS referral-rates whilst

For peer review only - http://bmjopen.bmj.com/site/about/guidelines.xhtml

reviewing the effectiveness of school-based interventions, and whether an increase or decrease in referrals would be observed. Considering the absence of reliable positive outcomes at the individual level at this point, a systemic perspective could be of value when considering any cost benefits to the wider health and social care services.

Further, it was unclear from the review what local or national political or strategic drivers instigated each study, and indeed, the extent to which children and young people were consulted in the process, design and delivery of the interventions. It was outside the scope of this review to explore the qualitative findings from the few studies that employed focus groups. Therefore, it is recommended that future qualitative reviews of school-based research are conducted in order to ensure that children's and young people's views as stakeholders in this work are sufficiently represented.

Limitations

This study was limited in its ability to source evaluations representative of the entire UK as the majority of studies were based in England. While efforts were made to source evaluations from elsewhere in the UK, the lack of validated measures or application of pre-post methodology meant that such evaluations from the 'grey literature' could not be included in this review. It should therefore be noted that there is much relevant work being conducted in schools across the UK. However, schools and local authorities should be urged to reliably evaluate their valuable efforts and contribute to the published literature, thereby demonstrating the important work being driven by teachers and policymakers nationwide.

This study was also limited in its date source in that only studies from the year 2000 were included in this review. While results from other systematic reviews suggested that little relevant research was done in the UK before this time, it could still be that some studies were missed due to this limit.

Implications

This review highlighted the need to employ robust methodological designs within school-based research in order for any effects to be interpreted meaningfully. Measurement issues exist where they do not adequately detect

For peer review only - http://bmjopen.bmj.com/site/about/guidelines.xhtml

change in universal populations, and there is a wide variety of measures used ranging from 'clinical' to wellbeing measures. This review concludes that school-based researchers across the UK should attempt to come together to discuss ways to address this issue and improve coherence in the literature.

An additional, imperative implication from this review is the proactive inclusion and involvement of teachers in this work. As has been commented elsewhere [45] without the 'buy-in' from teachers, any school-based intervention is less likely to sustain or achieve positive outcomes. In a time of additional pressures on teachers, the need to feel in control of initiatives is key. Of note, two of the studies in this review included adult-focussed exercises for the teachers themselves as an adjunct to the intervention training. This approach may go further to assist teachers' stress management and understanding of mental health whilst attending to the needs of their pupils.

Conclusions

The current evidence suggests there are neutral to small effects of universal, school-based interventions in the UK that aim to promote emotional or mental wellbeing or prevention of mental health difficulties. Whilst the real-world limitations of conducting research in schools exists, robust, long-term methodologies need to be attempted when conducting research in this area in order to explore the longitudinal impact of school-based interventions on wellbeing. Academic attainment, school attendance and rates of high-risk presentations also need to be further explored. This requires adequate recording of fidelity, the use of validated measures sensitive to mechanisms of change, reporting of those lost to follow up and any adverse effects, and the use of qualitative data to supplement quantitative outcomes. Interventions in the existing UK-based literature include educational, behavioural, cognitive and mindfulness components, each demonstrating variable results. Nevertheless, national and local policy [25, 26, 27, 46] indicates that there remains an appetite to develop work in this area in order to promote wellbeing outcomes for children and young people. In this case, further research collaborations are required across the UK to robustly demonstrate any benefits for pupils or on the wider system.

For peer review only - http://bmjopen.bmj.com/site/about/guidelines.xhtml

CONTRIBUTORSHIP

This piece of research was submitted in part fulfilment for a Doctorate degree in Clinical Psychology. KM (Dr Karen Mackenzie) was the main researcher and responsible for developing the research questions, conducting the search strategy and analysing results. CW (Professor Chris Williams) supervised this research and acted as co-rater during the search process.

ACKNOWLEDGEMENTS

Many thanks to Dr Claire Adey (CA) who assisted in the quality rating process.

COMPETING INTERESTS

CW is President of BABCP- the lead body for CBT in the UK, and a CBT researcher and trainer. He is also author of a range of CBT-based resources including some aimed at primary and secondary school populations. These are available commercially as books, online courses, and classes. He receives royalty, and is shareholder and director of a company that commercialises these resources.

FUNDING STATEMENT

This research received no specific grant from any funding agency in the public, commercial or not-for-profit sectors.

DATA SHARING

Supplementary data available upon request to the author.

FIGURE LEGEND

Figure 1: PRISMA flow diagram

Table 1: Overview of interventions

Table 2: Study design and outcomes – Primary schools

Table 3: Study design and outcomes – Secondary schools

For peer review only - http://bmjopen.bmj.com/site/about/guidelines.xhtml

REFERENCES

1. Kieling C, Baker-Heningham H, Belfer M, et al. Child and adolescent mental health worldwide: evidence for action. *Lancet* 2011;378:1515–25.

2. Costello EJ, Mustillo S, Erkanli A, et al. Prevalence and development of psychiatric disorders in childhood and adolescence. *Arch Gen Psychiatry* 2003; 60:837–44.

3. Kendall, P. C., Safford, S., Flannery-Schroeder, E., & Webb, A. Child anxiety treatment: Outcomes in adolescence and impact on substance abuse and depression at 7.4 year follow-up. *Journal of Consulting and Clinical Psychology* 2004;72:276–287.

4. Collins, K. A., & Dozois, D. J. A. What are the active ingredients in preventative interventions for depression? *Clinical Psychology: Science and Practice* 2008;15:313–330.

5. Patel V, Flisher AJ, Hetrick S, McGorry P. Mental health of young people: a global public-health challenge. *The Lancet* 2007;369(9569):1302–13.

6. World Health Organization. Atlas: Child and Adolescent Mental Health Resources: Global Concerns, Implications for the Future. WHO, 2005.

7. Masia-Warner, C., Nangle, D. W., & Hansen, D. J. Bringing evidence-based child mental health services to the schools: General issues and specific populations. *Education and Treatment of Children* 2006;29:165–172.

8. Domitrovich CE, Bradshaw CP, Greenberg MT, Embry D, Poduska JM, Ialongo NS. Integrated models of school-based prevention: logic and theory. *Psychology in Schools*. 2010;47(1):71–88.

9. Vostanis, P., Humphrey, N., Fitzgerald, N., Deighton, J., & Wolpert, M. (2013). How do schools promote emotional wellbeing among their pupils? Findings from a national scoping survey of mental health provision in English schools. *Child and Adolescent Mental Health* 2013;18:151–157.

For peer review only - http://bmjopen.bmj.com/site/about/guidelines.xhtml

10. Durlak, JA, Weissberg, RP, Dymnicki, AB, Taylor, RD & Schellinger, KB. The Impact of Enhancing Students' Social and Emotional Learning: A Meta-Analysis of School-Based Universal Interventions. *Child Development* 2011;82(1):405–432.

11. Wells, J, Barlow, J & Stewart-Brown. A systematic review of universal approaches to mental health promotion in school. *Health Education* 2003;103(4): 197-220.

12. Neil, A.L. & Christensen, H. Efficacy and effectiveness of school-based prevention and early intervention programs for anxiety. *Clinical Psychology Review* 2009; 29(3):208–215.

13. Mychailyszyn. M.P., Brodman, D.M., Read, K.L., et al. Cognitive Behavioural School-based interventions for anxious and depressed youth: a meta-analysis of outcomes. *Clinical Psychology Science and Practice* 2012;19:129-153.

14. Weare, K & Nind, M. Mental health promotion and problem prevention in schools: what does the evidence say? *Health Promotion International* 2011;26(1): 29-69.

15. Merry, S., Hetrick, S., Cox, G.R., et al. Psychological and educational interventions for preventing depression in children and adolescents. *Cochrane Database of Systematic Reviews* 2011;12.DOI:10.1002/14651858.CD003380.pub3.

16. Corrieri, S., Heider, D., Conrad, I., et al. School-based prevention programs for depression and anxiety in adolescence: a systematic review. Health Promotion International 2013;29(3):427-441.

17. Spence, S.H., & Shortt, A.L. Research review: Can we justify widespread dissemination of universal based interventions for the preventions of depression among children and adolescents? *Journal of Child Psychology and Psychiatry* 2007;48(6):526-542.

18. Calear, al. & Christensen, H. Review of internet-based prevention and treatment programs for anxiety and depression in children and adolescents. *The Medical Journal of Australia* 2010;192(11):12–14.

For peer review only

For peer review only - http://bmjopen.bmj.com/site/about/guidelines.xhtml

19. Kavanagh, J., Oliver, S., Lorenc, T., et al. School based cognitive-behavioural interventions: A systematic review of effects and inequalities. *Health Sociology Review* 2009;18: 61-78.

20. Stallard, P. School-based intervention for depression and anxiety in children and adolescents. *Evidence-based Mental Health* 2013;16(3):60-61.

21. Reavley, N., Bassilos, B., Ryan, S., et al. Interventions to build resilience in Young People: a literature review. *Victorian Health Promotion Foundation* 2015. Melbourne.

22. Sancassiani, F., Pintus, E., Holte, A,. et al. Enhancing the Emotional and Social Skills of the Youth to promote their Wellbeing and Positive Development: A systematic review of Universal School-based Randomised Controlled Trials. C*linical Practice & Epidemiology in Mental Health* 2015;11:21-40.

23. National Institute of Clinical Excellence. *Promoting children's social and emotional well-being in primary education*. 2008. Retrieved from http://www.nice.org.uk/guidance/index. Accessed June 2016.

24. Cheney, G., Schlosser, A., Nash, P., et al. Targeted group-based interventions in schools to promote emotional well-being: A systematic review. *Clinical Child Psychology & Psychiatry* 2014;19(3):412–438

25. Department of Education. *Mental health and behaviour in schools: Departmental advice for school staff*. 2016. www.nationalarchives.gov.uk/doc/open-government-licence/version/3. Accessed June 2016.

26. Education Scotland (no publication date). Curriculum for Excellence. *Health and Wellbeing Across Learning: Responsibilities for All. Experience and Outcomes*. www.educationscotland.gov.uk/health_wellbeing_experiences_outcomes_tcm4-540031.pdf Accessed June 2016.

27. CYMRU. 'Thinking positively: Emotional health and well-being in schools and Early Years settings'. 2010 www.wales.gov.uk/educationandskills. Accessed June 2016.

For peer review only - http://bmjopen.bmj.com/site/about/guidelines.xhtml

28. Downs, S. H., & Black, N. The feasibility of creating a checklist for the assessment of the methodological quality both of randomized and non-randomized studies of health care interventions. *Journal of Epidemiological Community Health* 1998;52:377–384.

29. Popay, J., H. Roberts, A. Sowden, F et al. *Guidance on the Conduct of Narrative Synthesis in Systematic Reviews: Final Report*. Swindon: ESRC Methods Programme 2006.

30. Attwood, M., Meadows, S., Stallard, P., et al. Universal and targeted computerised cognitive behavioural therapy (Think, Feel, Do) for emotional health in schools: Results from two exploratory studies. *Child and Adolescent Mental Health* 2012;17(3):173-178.

31. Berry. F., Axford, N., Blower, S., et al. The effectiveness and micro-costing analysis of a Universal, School based, Social-Emotional Learning programme in the UK: A cluster-randomised controlled trial. *School Mental Health* 2016;8:238-256.

32. Collins, S., Woolfson, L. M ., & Durkin, K. Effects on coping skills and anxiety of a universal school-based mental health intervention delivered in Scottish primary schools. *School Psychology International* 2013; 35(1):85-100.

33. Stallard, P., Simpson, N., Anderson, S., et al. The FRIENDS Emotional Health Programme: Initial Findings from a School-Based Project. *Child & Adolescent Mental Health* 2007;12(1):32-37.

34. Stallard, P., Skyrabina, E., Taylor, G., et al. Classroom-based cognitive behaviour therapy (FRIENDS): A cluster randomised controlled trial to Prevent Anxiety in Children through Education in Schools (PACES). *The Lancet Psychiatry* 2014;1(3):185-192.

35. Boniwell. I., Osin, E.N., & Martinez, C. Teaching Happiness at school: non-randomised controlled mixed-methods feasibility study on the effectiveness of well-being lessons. *The Journal of Positive Psychology* 2016;11(1):85-98.

36. Challen, A. R., Machin. S.J., & Gillham J.E. The UK Resilience Programme: a school-based universal nonrandomized pragmatic controlled trial. *Journal of Consulting & Clinical Psychology* 2014;82(1):75-89.

For peer review only - http://bmjopen.bmj.com/site/about/guidelines.xhtml

37. Chisholm, K. E., Patteron, P., Torgerson, C., et al. Impact of contact on adolescents' mental health literacy and stigma: the SchoolSpace cluster randomised controlled trial. *BMC Open* 2016,6:e009435.

38. Kuyken, W., Weare, K., Obioha, C., et al. Effectiveness of the Mindfulness in Schools Programme: Non-randomised controlled feasibility study. *The British Journal of Psychiatry* 2013; 203(2):126-131

39. Rice, F., Rawal, A., Riglin, L., et al. Examining reward-seeking, negative self-beliefs and over-general autobiographical memory as mechanisms of change in classroom prevention programs for adolescent depression. *Journal of Affective Disorders* 2015;186:320-327.

40. Naylor, P., Cowie, H.E., Watters, S.J., et al. Impact of a mental health teaching programme on adolescents. *The British Journal of Psychiatry* 2009;194:365-370.

41. Stallard. P., Phillips. R., Montgomery, A.A., et al. A cluster RCT to determine the clinical effectiveness and cost-effectiveness of classroom-based cognitive-behavioural therapy (CBT) in reducing symptoms of depression in high-risk adolescents. *Health Technology Assessment* 2013;*17*(47).

42. Erikson, Erik H. Identity, Youth and Crisis. New York: Norton. 1968.

43. Spear, L.P. Adolescent Neurodevelopment. *Journal of Adolescent Health* 2013; 52(2):S7-13. doi:10.1016/j.jadohealth.2012.05.006

44. Woolfson, R, Woolfson, L, Mooney, L, Bryce, D. Young people's views of mental health education in secondary schools: A Scottish study. *Journal of Child: care, health and development* 2008;35(6):790-798.

45. Weare, K. Editorial: Child and adolescent mental health in schools. *Child and Adolescent Mental Health* 2015;20(2):6-8.

46. Department for Education, Department of Health and Social Care. Transforming children and young people's mental health: a Green paper. December 2017. https://www.gov.uk/government/consultations/transforming-

For peer review only - http://bmjopen.bmj.com/site/about/guidelines.xhtml

children-and-young-peoples-mental-health-provision-a-green-paper accessed June 2018.

For peer review only

For peer review only - http://bmjopen.bmj.com/site/about/guidelines.xhtml

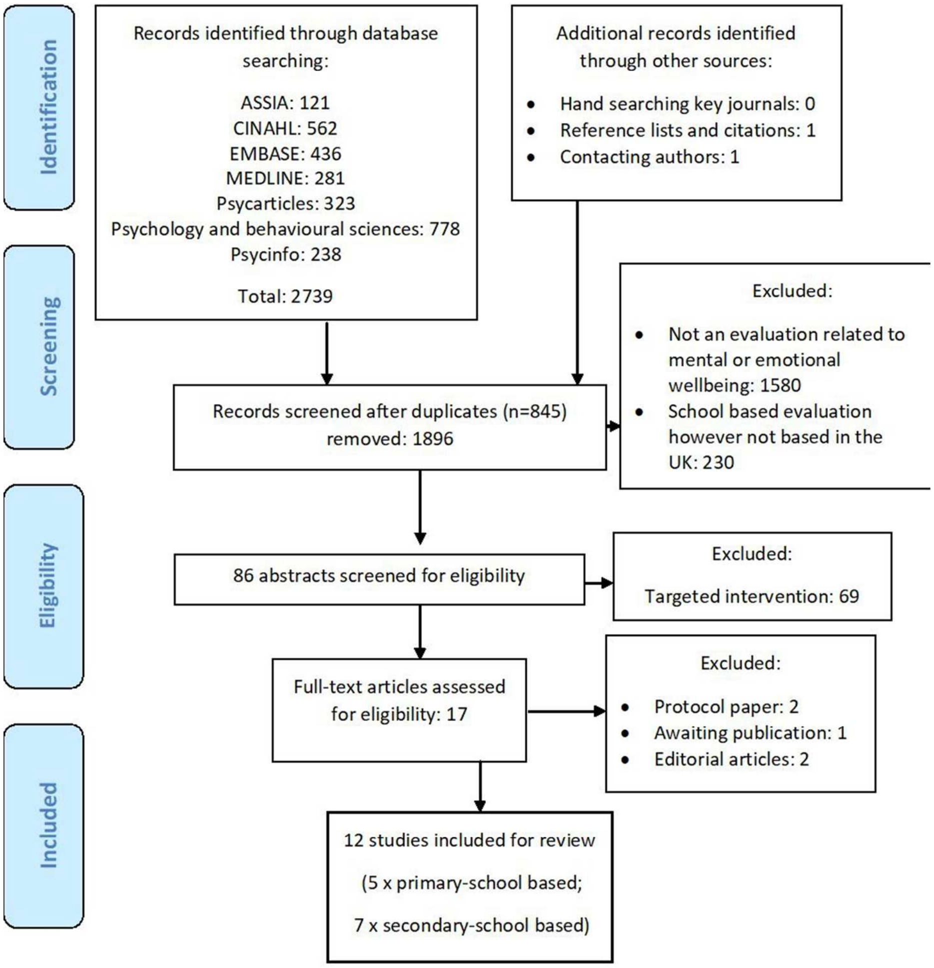

Figure 1: PRISMA diagram of systematic search process.

PRISMA diagram

83x107mm (300 x 300 DPI)

Search strategy database examples, including MeSH terms
(* indicates truncation of words).

ASSIA

((mental health) OR (mental health intervention OR early intervention)) AND ((school based) OR (scho* OR educat*))) AND (united kingdom OR sco* OR eng* OR northern Ir* OR wales OR wel* OR great britain)) AND (Adolescen* OR child* OR teen* OR youth OR young pe* OR pupil* OR student* OR learner* OR scho*)) AND (Anxi* OR depress* OR resilien* OR emotion* OR stress* OR psycho* OR wellbeing*)) AND (Evaluation OR (pilot OR trial) OR (comparison OR effective)) AND (yr(2000-2016) AND PEER(yes))) AND "SCHOOL BASED" or "SCHOOL"

EMBASE

adolescent health services; adolescent*; anxi*; based; brit*; child*; community mental health services; depress; depress *; early intervention (education); emotion* engl*; health promotion; ir*; kingdom; learner*; mental health; mental health services; northern; pe*; psycho*; pupil*; resilien*; scho*; school; school based; school health services; schools; scot*; stress*; student*; teen*; uk; wales; wel*; wellbeing*; young; young pe*;youth.

For peer review only

PRISMA 2009 Checklist

Section/topic	#	Checklist item	Reported on page #
**TITLE**			
Title	1	Identify the report as a systematic review, meta-analysis, or both.	1
**ABSTRACT**			
Structured summary	2	Provide a structured summary including, as applicable: background; objectives; data sources; study eligibility criteria, participants, and interventions; study appraisal and synthesis methods; results; limitations; conclusions and implications of key findings; systematic review registration number.	2
**INTRODUCTION**			
Rationale	3	Describe the rationale for the review in the context of what is already known.	5
Objectives	4	Provide an explicit statement of questions being addressed with reference to participants, interventions, comparisons, outcomes, and study design (PICOS).	6
**METHODS**			
Protocol and registration	5	Indicate if a review protocol exists, if and where it can be accessed (e.g., Web address), and, if available, provide registration information including registration number.	na
Eligibility criteria	6	Specify study characteristics (e.g., PICOS, length of follow-up) and report characteristics (e.g., years considered, language, publication status) used as criteria for eligibility, giving rationale.	7
Information sources	7	Describe all information sources (e.g., databases with dates of coverage, contact with study authors to identify additional studies) in the search and date last searched.	7-8
Search	8	Present full electronic search strategy for at least one database, including any limits used, such that it could be repeated.	Supp. file
Study selection	9	State the process for selecting studies (i.e., screening, eligibility, included in systematic review, and, if applicable, included in the meta-analysis).	8
Data collection process	10	Describe method of data extraction from reports (e.g., piloted forms, independently, in duplicate) and any processes for obtaining and confirming data from investigators.	8
Data items	11	List and define all variables for which data were sought (e.g., PICOS, funding sources) and any assumptions and simplifications made.	8
Risk of bias in individual studies	12	Describe methods used for assessing risk of bias of individual studies (including specification of whether this was done at the study or outcome level), and how this information is to be used in any data synthesis.	8
Summary measures	13	State the principal summary measures (e.g., risk ratio, difference in means).	8
Synthesis of results	14	Describe the methods of handling data and combining results of studies, if done, including measures of consistency (e.g., I^2) for each meta-analysis.	8

For peer review only - http://bmjopen.bmj.com/site/about/guidelines.xhtml

PRISMA 2009 Checklist

Section/topic	#	Checklist item	Reported on page #
Risk of bias across studies	15	Specify any assessment of risk of bias that may affect the cumulative evidence (e.g., publication bias, selective reporting within studies).	8
Additional analyses	16	Describe methods of additional analyses (e.g., sensitivity or subgroup analyses, meta-regression), if done, indicating which were pre-specified.	n/a
**RESULTS**			
Study selection	17	Give numbers of studies screened, assessed for eligibility, and included in the review, with reasons for exclusions at each stage, ideally with a flow diagram.	9
Study characteristics	18	For each study, present characteristics for which data were extracted (e.g., study size, PICOS, follow-up period) and provide the citations.	10-13
Risk of bias within studies	19	Present data on risk of bias of each study and, if available, any outcome level assessment (see item 12).	17-20
Results of individual studies	20	For all outcomes considered (benefits or harms), present, for each study: (a) simple summary data for each intervention group (b) effect estimates and confidence intervals, ideally with a forest plot.	17-20
Synthesis of results	21	Present results of each meta-analysis done, including confidence intervals and measures of consistency.	n/a
Risk of bias across studies	22	Present results of any assessment of risk of bias across studies (see Item 15).	n/a
Additional analysis	23	Give results of additional analyses, if done (e.g., sensitivity or subgroup analyses, meta-regression [see Item 16]).	n/a
**DISCUSSION**			
Summary of evidence	24	Summarize the main findings including the strength of evidence for each main outcome; consider their relevance to key groups (e.g., healthcare providers, users, and policy makers).	22-25
Limitations	25	Discuss limitations at study and outcome level (e.g., risk of bias), and at review-level (e.g., incomplete retrieval of identified research, reporting bias).	26
Conclusions	26	Provide a general interpretation of the results in the context of other evidence, and implications for future research.	26-27
**FUNDING**			
Funding	27	Describe sources of funding for the systematic review and other support (e.g., supply of data); role of funders for the systematic review.	28

From: Moher D, Liberati A, Tetzlaff J, Altman DG, The PRISMA Group (2009). Preferred Reporting Items for Systematic Reviews and Meta-Analyses: The PRISMA Statement. PLoS Med 6(7): e1000097. doi:10.1371/journal.pmed1000097

For more information, visit: **www.prisma-statement.org**.

For peer review only - http://bmjopen.bmj.com/site/about/guidelines.xhtml
